# Self-regulatory and metacognitive instruction regarding student conceptions: influence on students’ self-efficacy and cognitive load

**DOI:** 10.3389/fpsyg.2024.1450947

**Published:** 2024-10-22

**Authors:** Tim Hartelt, Helge Martens

**Affiliations:** Department of Biology Education, University of Kassel, Kassel, Germany

**Keywords:** self-regulated learning, metacognition, self-assessment, student conceptions, self-efficacy, cognitive load, science education

## Abstract

Self-regulatory and metacognitive instruction regarding student conceptions can help students become metacognitively (or more specifically, metaconceptually) aware of their conceptions and self-regulate their intuitive conceptions in scientific contexts when they are not appropriate. Two approaches have been found effective in enhancing conceptual knowledge: (a) self-assessing one’s conceptions and (b) acquiring conditional metaconceptual knowledge about why and in which contexts specific conceptions are appropriate or not. However, it is unclear how these approaches influence other cognitive and affective variables, such as self-efficacy and cognitive load. Nevertheless, it is essential to investigate whether making students aware of their intuitive conceptions affects their self-efficacy and to what extent reflecting on one’s conceptions requires additional (meta-)cognitive resources. Thus, we conducted an experimental intervention study using a 2×2 factorial design with *N* = 602 upper secondary biology students. Becoming metaconceptually aware of one’s (intuitive) conceptions did not lower students’ self-efficacy but enabled more accurate beliefs about their abilities. However, the self-assessment increased mental load, which partly suppressed the beneficial effect of the self-assessment on conceptual knowledge. In contrast, the instruction on conditional metaconceptual knowledge did not result in higher mental load and, thus, aligned more with students’ cognitive capacities. Furthermore, students with more pronounced general metaconceptual thinking reported lower mental load, implying that regular instruction focusing on metaconceptual thinking may reduce load. Thus, it is suggested to continuously promote students’ metaconceptual thinking and to embed metaconceptual activities (e.g., self-assessments) repeatedly across longer instructional units.

## 1 Introduction

Self-efficacy and cognitive load are essential factors when learners self-regulate and metacognitively plan, monitor, and evaluate their learning as they influence students’ effort, persistence, and ability to allocate (meta-)cognitive resources to challenging tasks (De Bruin et al., 2020; Panadero, 2017; Seufert, 2018; Wirth et al., 2020; Zimmerman, 2000). Thus, it is essential to investigate the influence of self-regulatory and metacognitive instructional approaches on self-efficacy and cognitive load. In science education, self-regulatory and metacognitive instruction can support students in becoming metacognitively aware of and self-regulate their intuitive conceptions of scientific topics. Explicitly addressing students’ intuitive conceptions is necessary as students frequently hold conceptions not in line with scientific concepts. These conceptions are often described as intuitive conceptions, preconceptions, alternative conceptions, and misconceptions (Maskiewicz and Lineback, 2013). Here, we will refer to intuitive conceptions as many student conceptions are based on cognitive biases, i.e., general, intuitive ways of thinking about the world (Coley et al., 2017; Coley and Tanner, 2015; Hartelt and Martens, 2024a; Richard et al., 2017). For example, students often intuitively explain scientific phenomena such as evolution as goal-directed or intentional (Aptyka et al., 2022; Bishop and Anderson, 1990; Pickett et al., 2022). Intuitive cognitive biases are pervasive, persistent, and their appropriateness depends on the specific context, i.e., they are appropriate in some contexts (e.g., everyday life) while not in others (e.g., scientific contexts; Coley et al., 2017; Coley and Tanner, 2015; Hartelt and Martens, 2024a; Shtulman and Calabi, 2012). It has been suggested that students should be enabled to reflect on their conceptions metacognitively and to self-regulate their intuitive conceptions in scientific contexts when they are not appropriate (González Galli et al., 2020; Hartelt and Martens, 2024a). Research indeed has shown that self-regulatory and metacognitive instruction can enhance conceptual knowledge of different science topics (Hartelt and Martens, 2024a; Kirbulut, 2012; Murtonen et al., 2018; Pickett et al., 2022; Wiser and Amin, 2001; Yürük et al., 2009). However, it is unclear how self-regulatory and metacognitive instruction regarding student conceptions affects students’ self-efficacy and cognitive load. Here, we investigate the effects of (a) a self-assessment of one’s conceptions and (b) instruction on conditional metaconceptual knowledge (i.e., metacognitive knowledge about why and in which contexts specific conceptions are appropriate or not) on self-efficacy and cognitive load.

## 2 Theoretical background

### 2.1 Self-regulatory and metacognitive instruction regarding student conceptions

Self-regulated learning and metacognition are important factors for learning. Consequently, both factors are also essential to effective science education (Gunstone and Mitchell, 2005; Zohar and Barzilai, 2013), especially for dealing with students’ intuitive conceptions of scientific topics (Hartelt and Martens, 2024a; Perez et al., 2022). Self-regulated learning and metacognition share a conceptual core, “that individuals make efforts to monitor their thoughts and actions and to act accordingly to gain some control over them” (Dinsmore et al., 2008, p. 404). According to Zimmerman and Moylan (2009), self-regulated learning “refers to self-generated thoughts, feelings, and actions attaining one’s learning goals” (p. 299), while metacognition as part of self-regulated learning “refers to knowledge, awareness, and regulation of one’s thinking” (p. 299). Besides metacognitive competencies, affective-motivational factors (e.g., self-efficacy) also play a role in models of self-regulated learning as students’ use of “metacognitive processes to learn is not merely a question of competence but is also a question of motivation to explain his or her willingness, effort, and persistence” (Zimmerman and Moylan, 2009, p. 299). In Zimmerman and Moylan’s (2009) cyclical, social cognitive model of self-regulated learning that is based on these assumptions, the authors propose three phases of self-regulated learning to which we relate the constructs of our study: (1) The forethought phase is composed of task analysis processes (e.g., analyzing the context of a task by drawing on conditional metaconceptual knowledge) and sources of self-motivation (e.g., self-efficacy beliefs), both influencing how a task is approached and how much (mental) effort is invested. (2) The performance phase involves self-control and self-observation aspects (e.g., monitoring one’s performance progress through recurring self-assessments). (3) The self-reflection phase consists of self-judgments (e.g., self-assessments comparing one’s performance with a standard) and the resulting self-reactions (e.g., continuing/modifying/avoiding further cycles of learning based on metacognitive evaluations and learning outcomes on affective variables such as self-efficacy). All these phases of self-regulated learning impose cognitive load in addition to the original learning task and, thus, are dependent on students’ (meta-)cognitive resources (Seufert, 2018).

In the context of conceptual learning, the term *metacognition* (i.e., thinking about one’s thinking in general) is often concretized and substituted by the term *metaconceptual* that is more specific as it refers to thinking about one’s conceptions in specific (Amin et al., 2014; Kirbulut et al., 2016; Yürük et al., 2009). The construct of metaconceptual thinking is not only highly relevant for the field of science education (e.g., Murtonen et al., 2018; Vosniadou, 1994; Wiser and Amin, 2001) but also for other educational contexts (e.g., other school subjects where developing conceptual knowledge is also a learning goal) and the field of educational psychology, as it concretizes general self-regulatory and metacognitive strategies for the acquisition of conceptual knowledge by considering students’ prior knowledge (i.e., their prior conceptions). Two subconstructs of metaconceptual thinking are proposed: metaconceptual awareness and metaconceptual regulation (Kirbulut et al., 2016). Metaconceptual awareness refers to one’s awareness about one’s understanding of a concept and the context in which a concept is used. Metaconceptual regulation refers to one’s monitoring of comprehension and one’s holding on to one’s or others’ conceptions. Metaconceptual awareness and regulation are positively related to conceptual knowledge (Alexander et al., 2006; Mason, 1994). Further, self-regulatory and metacognitive approaches that attempt to enhance students’ metaconceptual thinking are effective in fostering conceptual knowledge (Kirbulut, 2012; Murtonen et al., 2018; Pickett et al., 2022; Wiser and Amin, 2001; Yürük et al., 2009). Two approaches in particular have resulted in gains in conceptual knowledge: (1) a self-assessment of one’s conceptions and (2) instruction on conditional metaconceptual knowledge (Hartelt and Martens, 2024a).

Self-assessments can be differently defined and operationalized but are especially productive when they are operationalized as formative assessments of one’s learning processes and products providing self-generated feedback. In this operationalization, self-assessments are both instructional and self-regulatory processes and positively influence learning, performance, and metacognition, especially when the self-assessments are based on specific criteria (Andrade, 2019; Brown and Harris, 2013; Panadero and Alonso-Tapia, 2013; Panadero et al., 2023; Panadero and Romero, 2014; Sanchez et al., 2017). This way, students can metacognitively monitor their products/progress/conceptions against a standard that supports self-feedback (Rickey et al., 2023). While self-assessments have been related to several contents in various studies, only one recent study has investigated the effects of a self-assessment of one’s conceptions on conceptual knowledge (Hartelt and Martens, 2024a). Students were asked to self-assess their evolutionary explanations. For this, students used a self-assessment sheet that included intuitive and scientific conceptions as criteria. For example, one student correctly self-assessed an intuitive goal-directed conception in their explanation: “[..] Over time, the cheetah *had to* change in such a way that it became more competitive. This probably led to the body of the cheetah’s ancestor changing through mutations so that it could reach these higher speeds [..].” (italics represent the phrase that the student highlighted as a goal-directed conception; Hartelt and Martens, 2024b). A formative criteria-referenced self-assessment of one’s conceptions can, thus, enhance metaconceptual awareness of one’s intuitive and scientific conceptions and increase conceptual knowledge. Hartelt and Martens (2024a) reported that students who self-assessed their conceptions used more scientific conceptions afterward than students who did not self-assess their conceptions. However, no effect on students’ use of intuitive conceptions was found. Self-efficacy and cognitive load may be two variables helpful in explaining these findings and providing information on how self-assessments of one’s conceptions can be further adjusted to achieve even greater learning gains.

Another self-regulatory and metacognitive approach is instruction on conditional metaconceptual knowledge. Conditional knowledge can be defined as “knowing when and why to apply various actions” (Paris et al., 1983, p. 303). Previously, conditional knowledge has been linked to the use of reading and learning strategies (e.g., knowing when and why a specific reading strategy is appropriate or not). However, we suggest that conditional knowledge is also relevant in relation to the use of conceptions (i.e., knowing when and why to use which conceptions). The latter would be defined as conditional *metaconceptual* knowledge (Hartelt and Martens, 2024a). Acquiring conditional metaconceptual knowledge can support students in self-regulating their conceptions in a context-dependent manner. Self-regulating one’s conceptions in a context-dependent manner would mean to use intuitive conceptions in the context of everyday life where they may be appropriate but not in a scientific context where they may be inappropriate. For example, it is appropriate to explain human actions or the development of human-made artifacts in a teleological, goal-directed way but not scientific phenomena that do not happen due to a purpose or need (Coley and Tanner, 2012; González Galli et al., 2020; Hartelt and Martens, 2024a; Kampourakis, 2020). Instruction on conditional metaconceptual knowledge has been found effective in enhancing students’ conceptual knowledge: Students’ learning about the context-dependency of conceptions used fewer intuitive conceptions and more scientific conceptions in a scientific context (Hartelt and Martens, 2024a). However, it remains unclear how instruction on conditional metaconceptual knowledge and self-assessing one’s conceptions influence self-efficacy and cognitive load.

### 2.2 Self-efficacy

*Self-efficacy* is defined as “people’s beliefs about their capabilities to produce designated levels of performance” (Bandura, 1994, p. 71) and influences whether difficult tasks are executed or avoided, how much and how persistent effort is invested, and consequently how people perform on a task. According to Bandura (1994), there are four sources of self-efficacy: mastery experience, vicarious experiences, verbal persuasion, and physiological and affective states. Mastery experience (e.g., students’ prior experiences on a task) is the most influential one of these sources (Bandura, 1994; Dorfman and Fortus, 2019; Usher and Pajares, 2008): Success can strengthen self-efficacy beliefs whereas failure can undermine it. In contrast to self-assessments (a post-performance evaluation), self-efficacy is a pre-performance belief (that can be influenced by past performances).

#### 2.2.1 Relevance of self-efficacy in education

Self-efficacy is relevant in educational contexts because it is closely related to performance (Ardura and Galán, 2019; Kirbulut and Uzuntiryaki-Kondakci, 2019; Pintrich and De Groot, 1990). For example, self-efficacy correlates positively with the number of scientific conceptions one holds and negatively with the number of intuitive conceptions (Cakiroglu and Boone, 2002; Schoon and Boone, 1998; Hartelt and Martens, 2024c). Some theoretical considerations and empirical investigations suggest that students should have high self-efficacy because then they invest more effort in a task, persist in the face of challenges, and generally have a better learning outcome (Bandura, 1994; Bouffard-Bouchard et al., 1991; Hutchinson et al., 2008; Komarraju and Nadler, 2013; Sungur, 2007). However, self-efficacy unrealistically high compared to actual performance (e.g., high confidence despite one’s intuitive conceptions) can also result in students’ not being open to learning and reflecting on their conceptions because they do not see a necessity (Linnenbrink-Garcia et al., 2012; Maria, 1998; Pintrich, 1999; Talsma et al., 2019). Self-efficacy unrealistically low compared to actual performance, on the other hand, may be equally problematic because students may avoid learning and reflecting on their conceptions because they do not think they can be successful at this. Thus, in general, instructional materials should lead to accurately calibrated self-efficacy beliefs (Chen, 2003; Stankov and Lee, 2017), which can enable students to infer appropriate actions from their beliefs. However, students’ self-efficacy is often biased/miscalibrated, and students’ beliefs often exceed their capabilities (i.e., over-efficaciousness) or sometimes undercut their capabilities (i.e., under-efficaciousness; Chen and Zimmerman, 2007; Foster et al., 2017; Klassen, 2002; Osterhage et al., 2019; Talsma et al., 2019, 2020; Zimmerman et al., 2011). While over-efficaciousness may lead to superficial learning and, subsequently, poor performance, under-efficaciousness does not tend to be detrimental to performance outcomes (Chen, 2003; Talsma et al., 2019, 2020), even though the latter findings contradict theoretical assumptions (e.g., Bandura, 1994) and may need to be investigated further. In general, it is suggested that accurately calibrated beliefs about one’s abilities create greater potential for self-regulation as they reflect accurate metacognitive monitoring and enable metacognitive regulation of one’s cognition (Bol and Hacker, 2012; Hacker et al., 2008; Stone, 2000; Zimmerman and Moylan, 2009). Research indicates that more accurately calibrated beliefs about one’s abilities are associated with higher performance levels (Bol et al., 2005; Grimes, 2002; Hacker et al., 2000; Nietfeld et al., 2005; Prokop, 2020; Rutherford, 2017).

#### 2.2.2 Influence of self-regulatory and metacognitive instruction on self-efficacy

Theoretically and empirically, self-efficacy is closely related to self-regulated learning and metacognition. In different models of self-regulated learning, such as Zimmerman and Moylan’s (2009) one, self-efficacy beliefs (as part of a set of self-motivational beliefs) play a crucial role in the forethought phase and influence the activation and use of self-regulatory strategies, such as planning, monitoring, and evaluating in all three phases of self-regulated learning, i.e., the forethought, performance, and self-reflection phase (see also Efklides, 2011; Panadero, 2017; Panadero et al., 2017; Schunk and Ertmer, 2000). On the one hand, self-efficacy influences the amount and quality of self-regulatory and metacognitive strategies students use (Bouffard-Bouchard et al., 1991; Komarraju and Nadler, 2013; Pintrich and De Groot, 1990; Sungur, 2007; Zimmerman, 2000). On the other hand, self-regulatory and metacognitive interventions can positively influence students’ self-efficacy (Gentner and Seufert, 2020; Lavasani et al., 2011; Valencia-Vallejo et al., 2019). However, the cited interventions do not address students’ conceptions. When investigating the effectiveness of strategies dealing with student conceptions, most studies focus on cognitive outcome variables rather than affective ones, such as self-efficacy (Pacaci et al., 2023).

For self-regulatory and metacognitive interventions regarding student conceptions, it is assumed that those promote self-efficacy as “students are more likely to see the progress in their knowledge” when they are prompted “to review their ideas, to realize their conceptions, to monitor and evaluate their learning, and to compare their old and new understandings” (Kirbulut and Uzuntiryaki-Kondakci, 2019, p. 16). Indeed, metaconceptual awareness and regulation correlate positively with science self-efficacy (Kirbulut and Uzuntiryaki-Kondakci, 2019). However, correlational findings cannot provide causal evidence of whether instruction promoting metaconceptual thinking actually enhances self-efficacy, and how it affects self-efficacy bias. It could also be the case that becoming metaconceptually aware of one’s intuitive conceptions lowers self-efficacy because of awareness of one’s gaps in knowledge. Using a quasi-experimental research design, Kirbulut (2012) reported evidence that a teaching sequence including various self-regulatory and metacognitive instructional approaches regarding student conceptions (e.g., poster drawing, journal writing, and group discussions) affected students’ self-efficacy positively. However, the effects of specific approaches still need to be investigated through a controlled experimental design. We are not aware of any study investigating the effect of instruction on conditional metaconceptual knowledge on self-efficacy because this is a rather innovative instructional approach (see Hartelt and Martens, 2024a). However, numerous studies on the effects of self-assessments on self-efficacy exist, although none of these studies focused on student conceptions. Meta-analyses report positive effects of self-assessments (Panadero et al., 2017; Sitzmann et al., 2010), even though a recent meta-analysis found only an overall small effect (Panadero et al., 2023), and a narrative review also reports studies finding no effects (Panadero and Jonsson, 2013). Panadero et al. (2017) suggested that the reason for the generally positive effects on self-efficacy may be that students gain a deeper understanding of the task requirements, which may make students feel more confident; that they can observe changes in their competency through repeated self-assessments (thus, gaining a sense of mastery experience); and that they improve their performance, which in turn also enhances self-efficacy. However, when self-assessments are criteria-referenced, they may not enhance self-efficacy indiscriminately. Instead, they may enhance self-efficacy calibration because students become aware of the demands of a task and can better evaluate their abilities and conceptions metacognitively (see also Hartelt and Martens, 2024b). In relation to a self-assessment of one’s conceptions, self-efficacy could also be influenced negatively since becoming aware of one’s intuitive conceptions may be viewed as a failure (Perez et al., 2022; Perez and González Galli, 2024) and may lower one’s self-efficacy (for a negative correlation between intuitive conceptions and self-efficacy, see Hartelt and Martens, 2024c).

### 2.3 Cognitive load

*Cognitive load* is the “cognitive capacity which is used to work on a task” (Krell, 2017, p. 2). Its subconstruct mental load indicates the cognitive capacity necessary to process the complexity of a task (task-related), while mental effort reflects the cognitive capacity that is actually invested (subject-related; Krell, 2017; Paas and Van Merriënboer, 1994; Sweller et al., 2011). Both mental load and effort increase with increasing task complexity, even though the effect on mental load is higher (Krell, 2017). Mental load is negatively related to performance, meaning that people reporting a higher mental load perform worse on a task. The relationship between mental effort and performance is more inconclusive because people reaching the same performance level may need to work with different levels of mental effort (Krell, 2017; Minkley et al., 2018; Paas et al., 2003; Paas and Van Merriënboer, 1994).

#### 2.3.1 Relevance of cognitive load in education

Considering cognitive load induced by instructional approaches is relevant because learners’ capacity to process novel information is limited (Sweller et al., 1998). Thus, cognitive resources should be allocated to processes that are relevant to learning. Unnecessarily complex learning materials or instructional procedures, in contrast, may bind a lot of cognitive resources that cannot be used for learning the content, retaining the content in long-term memory, and metacognitively monitoring and self-regulating learning (Paas et al., 2003; Seufert, 2018; Sweller et al., 1998). While cognitive load can be positive when it is caused by processes conducive to learning, too high cognitive load (especially mental load) that is caused by processes not conducive to learning can hinder learning (Paas and Van Merriënboer, 1994; Sweller et al., 1998). Because the type and amount of cognitive load influence the effects on learning, it is essential to not only investigate effects of instructional approaches on cognitive load but also how cognitive load mediates the effects of instructional approaches on learning. Considering (the mediating effects of) cognitive load may be especially relevant when dealing with students’ intuitive conceptions because people hold on to their intuition when their information-processing resources are limited (Kelemen and Rosset, 2009; Kelemen et al., 2013; Rottmann et al., 2017; Shtulman and Valcarcel, 2012; Spatola and Chaminade, 2022). Thus, available cognitive resources are needed for students to organize their knowledge (i.e., differentiating between intuitive and scientific conceptions) and to resort to conceptions not based on intuitive reasoning when applying their knowledge.

#### 2.3.2 Influence of self-regulatory and metacognitive instruction on cognitive load

The interplay between cognitive load on one side and self-regulated learning and metacognition on the other side is complex (de Bruin et al., 2020; Seufert, 2018; Van Gog et al., 2011; Wirth et al., 2020): Consciously self-regulating and metacognitively planning/monitoring/evaluating one’s learning or conceptions requires additional (meta-)cognitive resources. Consequently, in all phases of self-regulated learning (i.e., in the forethought, performance, and self-reflection phase), further cognitive load is imposed by the self-regulation process in addition to the cognitive load imposed by the task itself (Seufert, 2018). However, self-regulative and metacognitive knowledge and strategies can also support completing complex tasks (e.g., because students know how to approach and chunk a task), thus reducing cognitive load. Consequently, previous studies have produced mixed results regarding effects of self-regulatory and metacognitive interventions on cognitive load (Chen et al., 2022; Glogger-Frey et al., 2016; López-Vargas et al., 2017; Moser et al., 2017; Zheng et al., 2019). The effect on cognitive load may depend on the characteristics of the specific type of intervention as well as on students’ preconditions, such as their prior conceptual knowledge (Dong et al., 2020; Kastaun et al., 2021; Seufert, 2018; Sweller et al., 1998) and their metaconceptual awareness and regulation. Thus, it seems necessary to consider several factors (i.e., external factors, such as instruction, and internal factors, such as students’ preconditions) to get a more extensive picture of the influencing variables on students’ cognitive load.

How self-regulatory and metacognitive approaches regarding student conceptions influence students’ cognitive load is still unclear. Studies investigating the effects of instructional materials addressing non-scientific conceptions showed no effect (Aptyka et al., 2022) or increased cognitive load (Muller et al., 2008; Poehnl and Bogner, 2013). Including intuitive, non-scientific conceptions in instructional materials may result in learners needing to process more information (compared to traditional instruction focusing on scientific conceptions only) and needing to delimit these intuitive conceptions from scientific conceptions, but further research is needed. While the novel approach of instruction on conditional metaconceptual knowledge has not yet been investigated in this regard, self-assessments have been found to potentially increase cognitive load (Fastré et al., 2012). However, probably because they scaffold the formation of self-assessments, providing students with criteria during self-assessment can reduce cognitive load in comparison with no provision of criteria (Krebs et al., 2022; however, for findings of increased stress, see Panadero and Romero, 2014). However, the task of self-assessing one’s intuitive and scientific conceptions may differ substantially from other self-assessing tasks because it is not only related to one’s learning products but also directly related to one’s cognition, which requires self-reflection on a deeper level. For example, it is also a challenge for teachers to assess students’ conceptions (Hartelt et al., 2022), suggesting it is difficult for students, too. Thus, the potentially complex task of self-assessing one’s conceptions could result in high cognitive load.

## 3 Current study and research questions

Using evolution as an exemplary topic where intuitive conceptions based on cognitive biases are frequent and persistent (Gregory, 2009), we investigated the effects of (a) a formative criteria-referenced self-assessment of one’s (intuitive and scientific) conceptions and (b) instruction on conditional metaconceptual knowledge on students’ self-efficacy and cognitive load. In prior analyses, both instructional approaches have proved to enhance students’ conceptual knowledge (Hartelt and Martens, 2024a), but their influence on students’ self-efficacy and cognitive load has not yet been investigated. In addition to investigating the main intervention effects on self-efficacy and cognitive load, we examined further questions relating to self-efficacy (RQ1–RQ2) and cognitive load (RQ3–RQ5) in connection with self-regulatory and metacognitive instruction regarding student conceptions.

### 3.1 RQs concerning self-efficacy

Regarding self-efficacy, we were not only interested in the effects of the instructional approaches on self-efficacy (RQ1) but also on self-efficacy bias (i.e., under-or over-efficaciousness; RQ2) because realistic self-efficacy is assumed to be preferable for further self-regulated learning (Chen, 2003; Stankov and Lee, 2017; Stone, 2000).

RQ1: To what extent do (a) a self-assessment and (b) instruction on conditional metaconceptual knowledge influence self-efficacy?

RQ2: To what extent do (a) a self-assessment and (b) instruction on conditional metaconceptual knowledge influence self-efficacy bias (i.e., under-or over-efficaciousness)?

### 3.2 RQs concerning cognitive load

In addition to the RQs regarding self-efficacy, we investigated the influence of the instructional approaches on cognitive load (RQ3). Further, we were also interested in whether cognitive load experienced during the instruction influenced learning positively or negatively (RQ4). In a further, more explorative analysis, we examined to what extent students’ preconditions (i.e., prior conceptual knowledge and general metaconceptual awareness and regulation) influenced their cognitive load (RQ5). These analyses can provide suggestions on how to implement self-regulatory and metacognitive instruction regarding student conceptions to fit with students’ preconditions (i.e., to achieve a suitable level of cognitive load).

RQ3: To what extent do (a) a self-assessment and (b) instruction on conditional metaconceptual knowledge influence cognitive load?

RQ4: To what extent are the effects of interventions (a) and (b) on conceptual knowledge mediated by cognitive load?

RQ5: To what extent do students’ preconditions (i.e., prior conceptual knowledge and general metaconceptual awareness and regulation) influence their cognitive load during the interventions (a) and (b)?

## 4 Methods

### 4.1 Participants

We conducted a study with a 2×2 factorial, pre-post-follow-up test design in upper secondary level biology classes in Germany. *N* = 602 students participated in the study from the pre-to post-test (participants who missed one of the lessons that were part of the study were excluded from all analyses). *n* = 400 students also completed the follow-up questionnaire. Drop-out from post-to follow-up test was random and evenly distributed across the groups [31–35%], among others, due to Covid-related quarantines. Students were, on average, *M* = 17.29 years old. They attended classes 10 to 13 (class 10: 9%; class 11: 21%; class 12: 38%; class 13: 31%). 34% identified as male, 65% as female, and 2% as diverse. On average, students reported having had *M* = 9.92 prior evolution lessons in upper secondary level (for an exhaustive description of the sample, see Supplementary Table S1).

### 4.2 Study design

**Figure 1:**
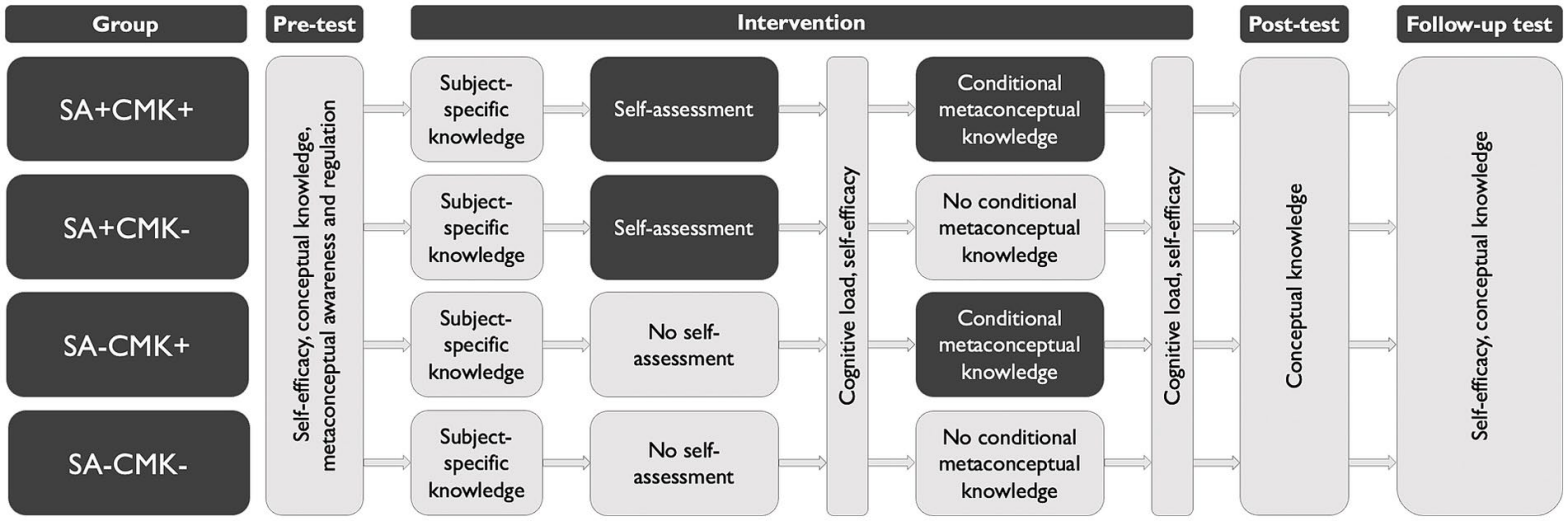
Study design of the experimental intervention study. Subject-specific knowledge was taught using an interactive simulation on natural selection (Hartelt and Martens, 2024d). The follow-up test was conducted 6 to 11 weeks (*M* = 8.27) after the post-test. SA = intervention on self-assessment; CMK = instruction on conditional metaconceptual knowledge; plus sign (+) = the group received the respective intervention; minus sign (−) = the group did not receive the respective intervention.

Within our study with a 2×2 factorial, pre-post-follow-up test design (see Figure 1, Table 1), all students worked individually (i.e., without interaction with their classmates or teachers) on the digital intervention materials (see 4.2.1–4.2.2) and questionnaires (see 4.3) that were provided in the learning management system *Moodle*. Students were randomly assigned (simple random assignment) to four different groups (group SA + CMK+: *n* = 153; SA + CMK-: *n* = 144; SA-CMK+: *n* = 158; SA-CMK-: *n* = 147; SA = intervention on self-assessment; CMK = instruction on conditional metaconceptual knowledge; plus sign (+) = the group received the respective intervention; minus sign (−) = the group did not receive the respective intervention).

**Table 1:** Overview of the interventions and measures.

Interventions	Description
Instruction for all students: Subject-specific knowledge	Students of all groups worked on an interactive simulation with explicit reference to the scientific key concepts of evolution through natural selection (Hartelt and Martens, 2024d)
Intervention (a): Self-assessment (SA+)	Students of the respective intervention groups (SA + CMK+ and SA + CMK-) conducted a formative criteria-referenced self-assessment of their intuitive and scientific conceptions of evolution in their prior explanation of evolution
Intervention (b): Conditional metaconceptual knowledge (CMK+)	Students of the respective intervention groups (SA + CM+ and SA-CMK+) received instruction on the context-dependency of intuitive conceptions (i.e., everyday versus scientific context, different scientific contexts, and different social contexts)
Measured variables	Description
Self-efficacy	Students’ beliefs about their abilities regarding explaining evolutionary changes (SE^3^ instrument; *Self-Efficacy Regarding Explaining Evolutionary Changes*; Hartelt and Martens, 2024c)
Self-efficacy bias	Under-or over-efficaciousness in relation to the average student in the pre-test (calculated by using the scores of students’ self-efficacy and conceptual knowledge at the respective measuring points)
Mental load	Cognitive capacity that was necessary to process the complexity of the interventions (task-related cognitive load; StuMMBE-Q; *Students’ Mental Load and Mental Effort in Biology Education-Questionnaire*; Krell, 2017)
Mental effort	Students’ actually invested cognitive capacity in the interventions (subject-related cognitive load; StuMMBE-Q; Krell, 2017)
Conceptual knowledge	Students’ use of intuitive and scientific conceptions when explaining evolutionary changes (ACORNS; *Assessing COntextual Reasoning about Natural Selection*; Nehm et al., 2012)
Metaconceptual awareness	Students’ metacognitive awareness of their conceptual thinking (MARS; *Metaconceptual Awareness and Regulation Scale*; Kirbulut et al., 2016)
Metaconceptual regulation	Students’ metacognitive regulation of their conceptual thinking (MARS; Kirbulut et al., 2016)

Before the interventions, students in all groups worked on an interactive simulation (Hartelt and Martens, 2024d)^1^ that focuses on seven scientific key concepts of natural selection (Nehm et al., 2010): within-species variation, heritability of variation, differential survival/reproduction of individuals, overproduction of offspring, resource limitation, competition, and generational changes in the distribution or frequency of variation. Thus, the interactive simulation is intended to foster subject-specific knowledge. During the interactive simulation, students observed the evolutionary changes of a model population. They participated in several activities that influenced the outcome of the simulation (e.g., rolling the dice decided whether random mutations occurred and potentially increased in frequency in the population over generations). The interactive simulation did not address students’ intuitive conceptions and did not intend to enhance or provoke metaconceptual knowledge or processes. Instead, the scientific knowledge acquired through the simulation should provide a basis for the subsequent metaconceptual interventions. To systematically investigate the effects of (a) a self-assessment and (b) instruction on conditional metaconceptual knowledge, the interactive simulation was not an independent variable manipulated within our study design and was identical for all students. Consequently, we will not focus on the effects of the interactive simulation.

#### 4.2.1 Intervention (a): formative criteria-referenced self-assessment of one’s (intuitive and scientific) conceptions

**Figure 2:**
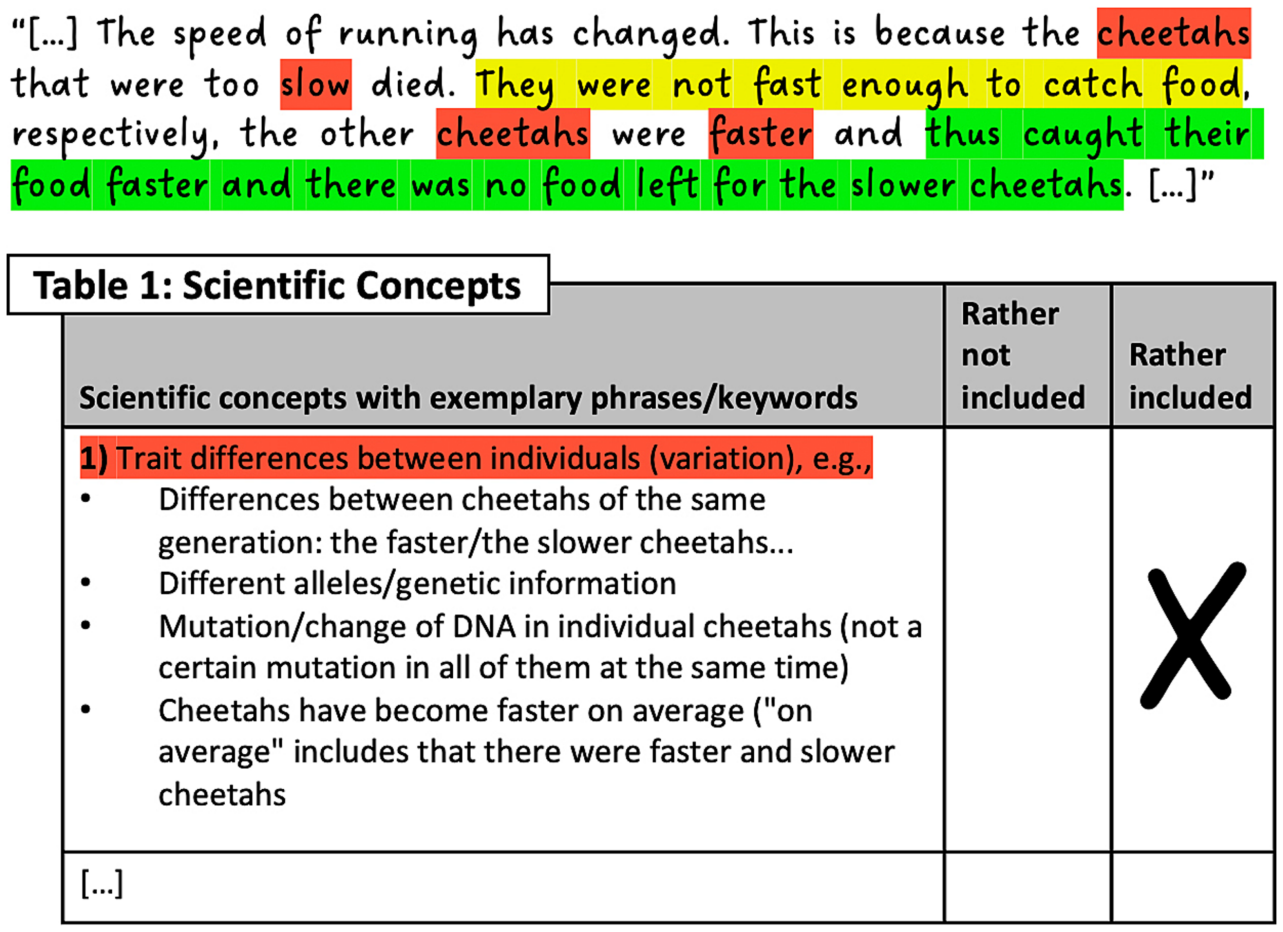
Simplified illustration of the self-assessment procedure regarding one of the 10 conceptions that should be self-assessed (in total, seven scientific key concepts and three intuitive conceptions based on cognitive biases). Red, self-assessed within-species variation; yellow, self-assessed resource limitation; green, self-assessed competition.

Students in the intervention groups (SA + CMK+, SA + CMK-) received a self-assessment sheet with the seven scientific key concepts of natural selection (see also 4.2) and three categories of intuitive conceptions based on cognitive biases (Coley and Tanner, 2015; Gregory, 2009; Nehm et al., 2010): teleology (goal-directed, purposeful changes), anthropomorphism (self-awareness and intentional changes of individuals/species), and essentialism (gradual transformation of the entire species by neglecting the existence and/or relevance of variation). In the self-assessment sheet, categories of intuitive and scientific conceptions were supplemented by short explanations and examples. The students should examine their prior explanation of evolution (provided by the students after the pre-test and before the interactive simulation) by color-coding in their text and ticking off in the list the conceptions they have used (for an example, see Figure 2; for the materials, see also Hartelt and Martens, 2025). The control groups (SA-CMK+, SA-CMK-) also received the list of scientific conceptions but were not explicitly asked to self-assess their explanation. Instead, they highlighted and explained important scientific terms in the list and, thus, engaged with subject-specific content instead of their conceptions.

#### 4.2.2 Intervention (b): instruction on conditional metaconceptual knowledge

The instruction on conditional metaconceptual knowledge (groups SA + CMK+, SA-CMK+) aimed to raise awareness about how the appropriateness of intuitive conceptions based on cognitive biases depends on the context. The instruction focused on three cognitive biases: teleology, anthropomorphism, and essentialism (see also 4.2.1). Further, it was differentiated between the everyday versus scientific context, different scientific contexts, and different social contexts. For example, students received information on why teleological thinking may be valuable in everyday life where we as humans act in a goal-directed way but problematic in the context of evolution where species do not change in a goal-directed way but through natural processes. Students read informational texts regarding the context-dependency of conceptions and worked on different tasks. For example, they were asked to decide whether certain statements based on intuitive cognitive biases are appropriate depending on a given context. For instance, the statement “He [i.e., a person] exercises in order to get bigger muscles.” would be an appropriate goal-directed explanation in an everyday context. In contrast, the statement “The virus mutated in order to become more contagious and, thus, spread better.” would be an inappropriate goal-directed explanation in the context of evolution. By focusing on the context-dependency of conceptions in the intervention materials, students’ intuitive conceptions were valued, e.g., as useful in everyday life (for the materials, see also Hartelt and Martens, 2025). In the control groups (SA + CMK-, SA-CMK-), conditional metaconceptual knowledge was not promoted, and students acquired more subject-specific knowledge related to evolution. Consequently, they received more instruction on subject-specific knowledge than the intervention groups.

### 4.3 Instruments and measures

#### 4.3.1 Self-efficacy and self-efficacy bias

We measured self-efficacy at four time points (pre-test, after intervention [a], after intervention [b], and follow-up test) with the SE^3^ instrument (*Self-Efficacy Regarding Explaining Evolutionary Changes*; Hartelt and Martens, 2024c). This instrument consists of eight items scored on a six-point Likert scale (for all measuring points: *ω* ≥ 0.913; very high reliability), e.g., “I can use appropriate terminology to explain evolutionary changes even if I cannot look up scientific terms once more.” We used the SE^3^ instrument because it measures self-efficacy on a task-specific level corresponding to the specificity level of the intervention and other measurement instruments used (e.g., the instrument measuring conceptual knowledge, see 4.3.3). Validity evidence based on test content, response processes, internal structure, and relations to other variables has been provided for the SE^3^ instrument (Hartelt and Martens, 2024c; see also Stolz, 2021).

Self-efficacy bias was operationalized as under- or over-efficaciousness in relation to the average student in the pre-test. Thus, self-efficacy bias was calculated for individual measuring points by subtracting the respective z-standardized NSPQ score (natural selection performance quotient, representing students’ conceptual knowledge; see 4.3.3) from the respective z-standardized self-efficacy score. Regardless of the measuring point, we used means and standard deviations of the pre-test for z-standardization of the NSPQ and self-efficacy scores, thus setting the average student pre-test student as a baseline for comparison. Consequently, negative values tend to reflect under-efficaciousness, and positive values tend to reflect over-efficaciousness in relation to the average pre-test student.

#### 4.3.2 Cognitive load

To measure cognitive load directly after each of both interventions (a and b), we used the StuMMBE-Q (*students’ Mental Load and Mental Effort in Biology Education-Questionnaire*; Krell, 2017). This instrument consists of 6 items for both the subscale of mental load (e.g., “The tasks were challenging.”) and mental effort (e.g., “I have given my best to solve the tasks.”). Responses were given on a seven-point Likert scale (for all measuring points: mental effort: *ω* ≥ 0.854; mental load: *ω* ≥ 0.891; high reliability). Krell (2015, 2017) investigated and provided validity evidence based on test content, internal structure, and relations to other variables for this instrument.

#### 4.3.3 Conceptual knowledge

We measured conceptual knowledge in the pre-test, post-test (i.e., after completing both interventions [a] and [b]), and follow-up test using the ACORNS instrument (*Assessing COntextual Reasoning about Natural Selection*; Nehm et al., 2012) for which validity evidence based on relations to other variables (especially convergent validity) has been reported (Beggrow et al., 2014; Nehm et al., 2012). The ACORNS instrument is frequently used to investigate students’ conceptual knowledge about evolution (e.g., Aptyka et al., 2022; Federer et al., 2015; Großschedl et al., 2018; Ha et al., 2019; Pobiner et al., 2018; Sbeglia and Nehm, 2024). We used two open-response ACORNS items at each measuring point. Students should explain the evolutionary changes of two different animal species, e.g., “How would a biologist explain how the long tongue evolved in anteaters when their ancestors had a shorter tongue?” (For the other items, see Hartelt and Martens, 2024a). We calculated students’ use of seven scientific key concepts (Nehm et al., 2010; sum score for both items: 0–14) and three intuitive conceptions based on cognitive biases (Nehm et al., 2010; sum score for both items: 0–6). The rating was conducted by two raters and interrater reliability was high (*ĸ* = 0.855). Furthermore, we calculated the NSPQ that quantifies students’ conceptual knowledge in a single score (0–1) by considering the ratio between key concepts and intuitive conceptions based on cognitive biases: Higher numbers of key concepts increase the NSPQ whereas higher numbers of intuitive conceptions based on cognitive biases decrease it (for a detailed description of the calculation of the NSPQ, see Supplementary Table S2). While we consider it important to differentiate in most of the analyses between key concepts and intuitive conceptions based on cognitive biases (i.e., using two different scores for conceptual knowledge), the NSPQ (i.e., a single score for conceptual knowledge) allows calculations for which it is not meaningful to differentiate (i.e., calculating self-efficacy bias; see 4.3.1).

#### 4.3.4 Metaconceptual awareness and regulation

For measuring students’ self-reported metaconceptual awareness and regulation in the pre-test, we used the MARS (*Metaconceptual Awareness and Regulation Scale*; Kirbulut et al., 2016; for the adapted and translated version used in this study, see Hartelt and Martens, 2024a). This instrument consists of 10 items scored on a six-point Likert scale for the two factors Metaconceptual Awareness (e.g., “I know what I did *not* understand about a biological topic.”; 4 items; *ω* = 0.708) and Metaconceptual Regulation (e.g., “I evaluate whether the ideas coming from my friends, my teacher, or other sources [book, journal, etc.] related to a biological topic are plausible or not.”; 6 items; *ω* = 0.741; acceptable reliability). Validity evidence based on test content, response processes, internal structure, and relations to other variables has been reported for the original instrument (Kirbulut et al., 2016).

### 4.4 Data analysis

We used SPSS IBM Statistics (version 28.0.1.0) for all statistical analyses. In case of missing data, pairwise deletion was performed for individual analyses. To investigate intervention effects on students’ self-efficacy, we calculated a mixed ANOVA with the different intervention groups (i.e., SA + CMK+, SA + CMK-, SA-CMK+, and SA-CMK-) as between-group factor and time (pre-test, after intervention [a], after intervention [b], and follow-up test) as within-group factor. To investigate intervention effects on students’ self-efficacy bias, we proceeded congruently but only used three measuring points because self-efficacy bias could not be calculated after intervention (a) as students’ conceptual knowledge was not measured at this point. To investigate intervention effects on students’ cognitive load, we performed univariate ANOVAs for students’ mental effort and mental load after both the intervention (a) with/without self-assessment and the intervention (b) with/without instruction on conditional metaconceptual knowledge. To investigate whether cognitive load influenced the effects of the interventions (a) and (b) on conceptual knowledge, we conducted mediation analyses with 95% confidence intervals from 1,000 bootstrap samples together with heteroscedasticity consistent standard errors using the SPSS macro PROCESS v4.3 (Hayes, 2023). To investigate the effects of students’ preconditions on students’ cognitive load during the interventions, we calculated two-tailed Spearman correlations between students’ cognitive load (i.e., mental effort and mental load) on the one side and students’ preconditions (i.e., prior conceptual knowledge and metaconceptual awareness and regulation) on the other side.

## 5 Results

### 5.1 Baseline description

We found no significant between-group differences in pre-test scores of dependent variables or demographic variables, demonstrating successful random allocation of the students to the different groups (for descriptive statistics of the individual groups and the total sample, see Supplementary Table S1).

### 5.2 RQs concerning self-efficacy

#### 5.2.1 RQ1: intervention effects on self-efficacy

**Figure 3:**
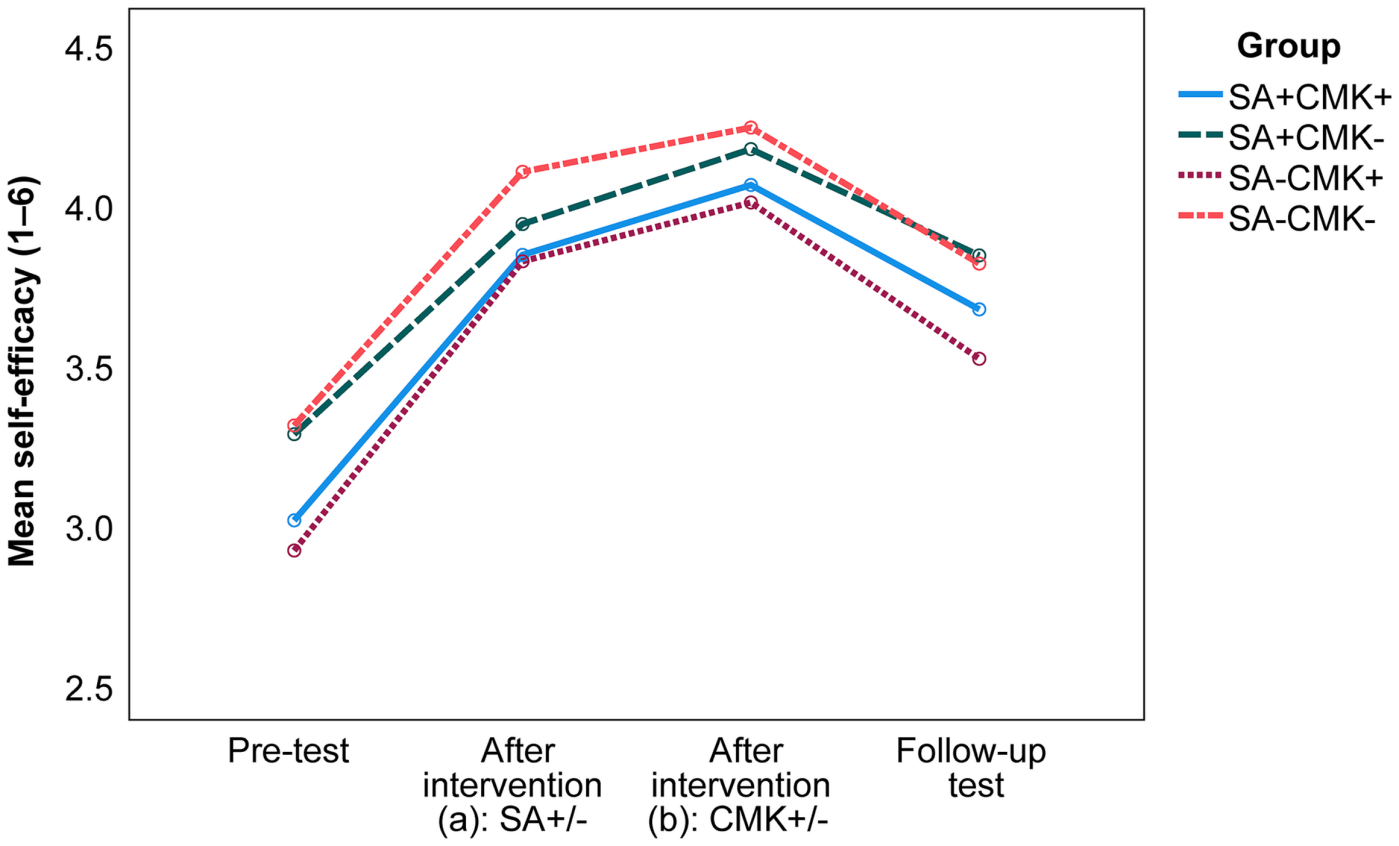
Development of students’ self-efficacy (mean scores) over the different measuring points as a function of group allocation. For descriptive statistics, see Supplementary Table S3. SA = intervention on self-assessment; CMK = instruction on conditional metaconceptual knowledge; plus sign (+) = the group received the respective intervention; minus sign (−) = the group did not receive the respective intervention.

Regarding self-efficacy (see Figure 3), we calculated a mixed ANOVA and found no interaction effect between time and intervention group, Greenhouse–Geisser *F* (7.684, 865.681) = 0.734, *p* = 0.656. No main effect for group was found, meaning that the intervention groups did not differ significantly [*F* (3, 338) = 2.526, *p* = 0.057]. However, there was a main effect for time, Greenhouse–Geisser *F* (2.561, 865.618) = 167.101, *p* < 0.001, $\eta_{p}^{2}$ = 0.331 (large effect; Cohen, 1988). Bonferroni *post hoc* tests revealed differences in self-efficacy between all four measuring points (all *p*s < 0.001). Self-efficacy increased across all groups from the pre-test to after intervention (a) with/without self-assessment (*M*_Diff_ = 0.796), further increased to after intervention (b) with/without instruction on conditional metaconceptual knowledge (*M*_Diff_ = 0.194), and decreased to the follow-up test (*M*_Diff_ = −0.409) but still remained above the pre-test level (*M*_Diff_ = 0.581).

#### 5.2.2 RQ2: intervention effects on self-efficacy bias

**Figure 4:**
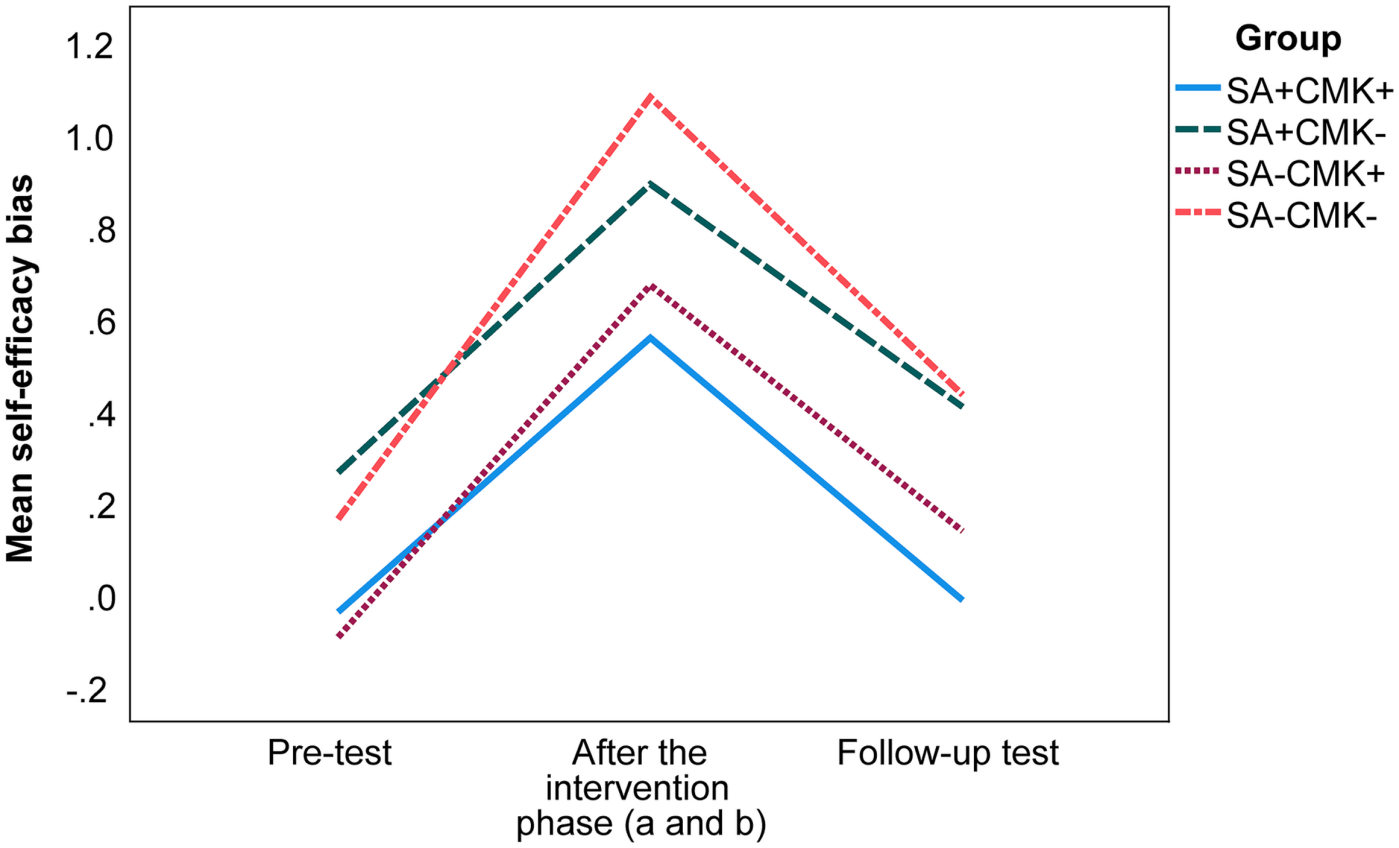
Development of students’ self-efficacy bias (mean scores) over the different measuring points as a function of group allocation. For descriptive statistics, see Supplementary Table S4. Values above zero reflect over-efficaciousness and values below zero reflect under-efficaciousness (in comparison to average pre-test efficaciousness). Self-efficacy bias was only determined for the three measurement points depicted above but not after intervention (a) because after intervention (a), only self-efficacy was measured but not conceptual knowledge. SA = intervention on self-assessment; CMK = instruction on conditional metaconceptual knowledge; plus sign (+) = the group received the respective intervention; minus sign (−) = the group did not receive the respective intervention.

Regarding self-efficacy bias (see Figure 4), we calculated a mixed ANOVA and found no interaction effect between time and intervention group [*F* (6, 716) = 0.773, *p* = 0.591]. However, there was a main effect for time [*F* (2, 716) = 70.570, *p* < 0.001, $\eta_{p}^{2}$ = 0.165; large effect; Cohen, 1988]. Bonferroni *post hoc* tests showed differences in self-efficacy bias between all three measuring points (all *p*s ≤ 0.032). Over-efficaciousness increased across all groups from the pre-test to the post-test (*M*_Diff_ = 0.726) and decreased to the follow-up test (*M*_Diff_ = −0.559) but remained above the pre-test level (*M*_Diff_ = 0.167). Further, we found a main effect for group [*F* (3, 358) = 5.217, *p* = 0.002, $\eta_{p}^{2}$ = 0.042; small effect; Cohen, 1988]. In the post-test, there was a difference between groups SA + CMK+ and SA-CMK- (*M*_Diff_ = 0.523; *p* = 0.007), meaning that students receiving both self-regulatory and metacognitive interventions (SA + CMK+) developed a less pronounced over-efficaciousness compared to students receiving no self-regulatory and metacognitive intervention (SA-CMK-; for other group comparisons, all *p*s ≥ 0.067). In the follow-up test, group SA + CMK+ again had a lower self-efficacy bias (respectively, no noteworthy self-efficacy bias) compared to groups SA-CMK- (*M*_Diff_ = 0.447; *p* = 0.023) and SA + CMK- (*M*_Diff_ = 0.420; *p* = 0.041) with a higher over-efficaciousness (for other group comparisons, all *p*s ≥ 0.320), revealing that instruction on conditional metaconceptual knowledge reduced self-efficacy bias in the follow-up test.

### 5.3 RQs concerning cognitive load

#### 5.3.1 RQ3: intervention effects on cognitive load

**Figure 5:**
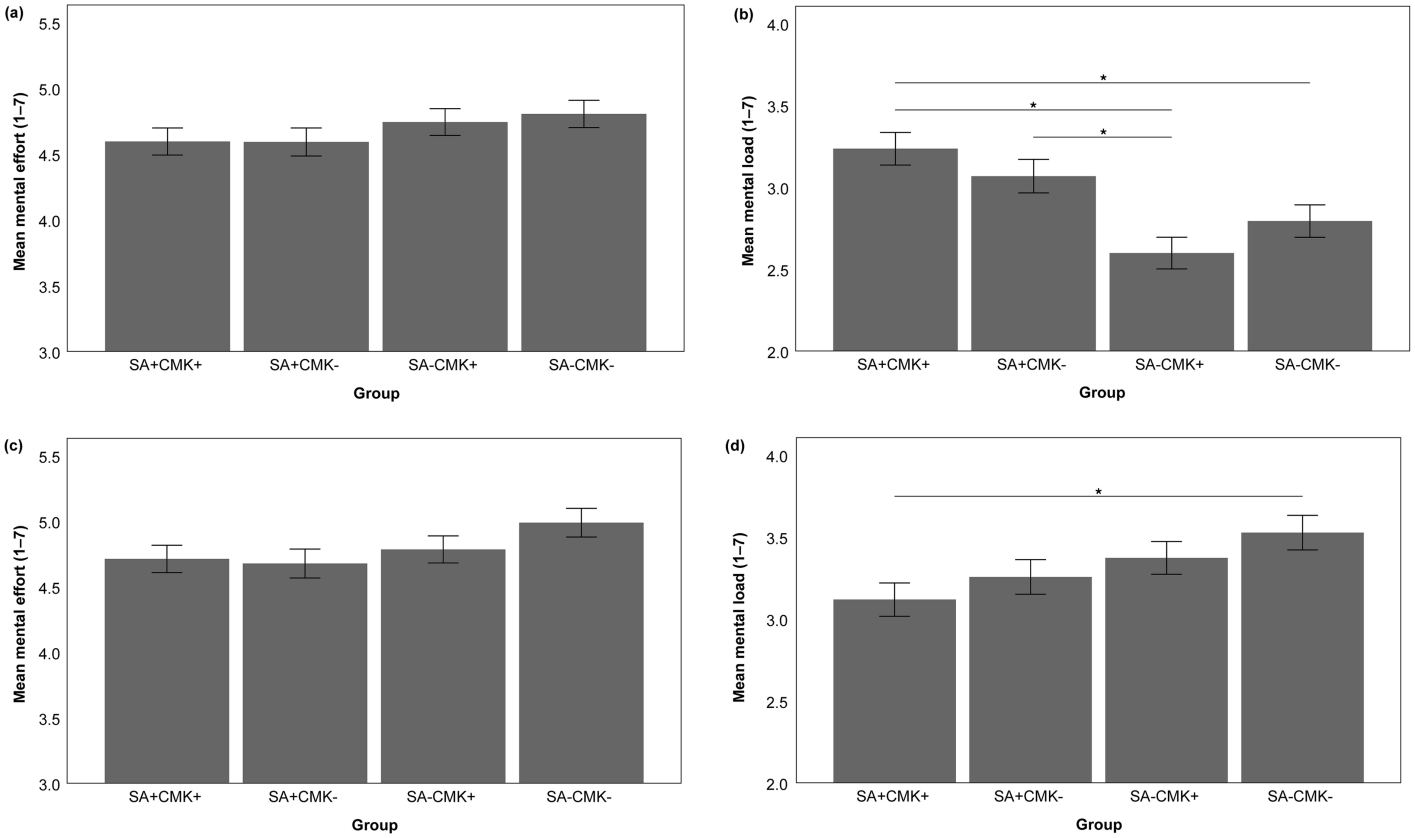
Students’ cognitive load related to the interventions. **(a)** Mental effort related to the intervention **(a)** with/without SA. **(b)** Mental load related to the intervention **(a)** with/without SA. **(c)** Mental effort related to the intervention **(b)** with/without CMK. **(d)** Mental load related to the intervention **(b)** with/without CMK. For descriptive statistics, see Supplementary Table S5. Mean scores are displayed in the figure, and error bars represent standard errors; * = *p* < 0.05. SA = intervention on self-assessment; CMK = instruction on conditional metaconceptual knowledge; plus sign (+) = the group received the respective intervention; minus sign (−) = the group did not receive the respective intervention.

After completing the intervention (a) with/without self-assessment, there was no difference between the groups in their mental effort [*F* (3, 571) = 1.064, *p* = 0.364; univariate ANOVA; see Figure 5a]. However, there was a difference in their mental load [*F* (3, 573) = 8.274, *p* < 0.001, $\eta_{p}^{2}$ = 0.042; medium effect; Cohen, 1988; univariate ANOVA; see Figure 5b]. Gabriel’s *post hoc* tests revealed higher mental load for group SA + CMK+ compared to group SA-CMK+ (*M*_Diff_ = 0.637; *p* < 0.001), higher mental load for group SA + CMK+ compared to group SA-CMK- (*M*_Diff_ = 0.442; *p* = 0.010), and higher mental load for group SA + CMK- compared to group SA-CMK+ (*M*_Diff_ = 0.470; *p* = 0.005; for all other *post hoc* tests, *p*s ≥ 0.286). Thus, the self-assessment resulted in a higher mental load than the control materials.

After completing the intervention (b) with/without instruction on conditional metaconceptual knowledge, the groups again did not report different levels of mental effort [*F* (3, 563) = 1.631, *p* = 0.181; univariate ANOVA; see Figure 5c], but different levels of mental load [*F* (3, 565] = 2.794, *p* < 0.040, $\eta_{p}^{2}$ = 0.015; small effect; Cohen, 1988; univariate ANOVA; see Figure 5d). Gabriel’s *post hoc* tests showed that groups SA + CMK+ and SA-CMK- differed (*M*_Diff_ = −0.408; *p* = 0.033), with the group receiving both self-regulatory and metacognitive interventions (SA + CMK+) reporting lower mental load than the group receiving no self-regulatory and metacognitive intervention (SA-CMK-; for all other *post hoc* tests, *p*s ≥ 0.339).

#### 5.3.2 RQ4: mediating effects of cognitive load

**Figure 6:**
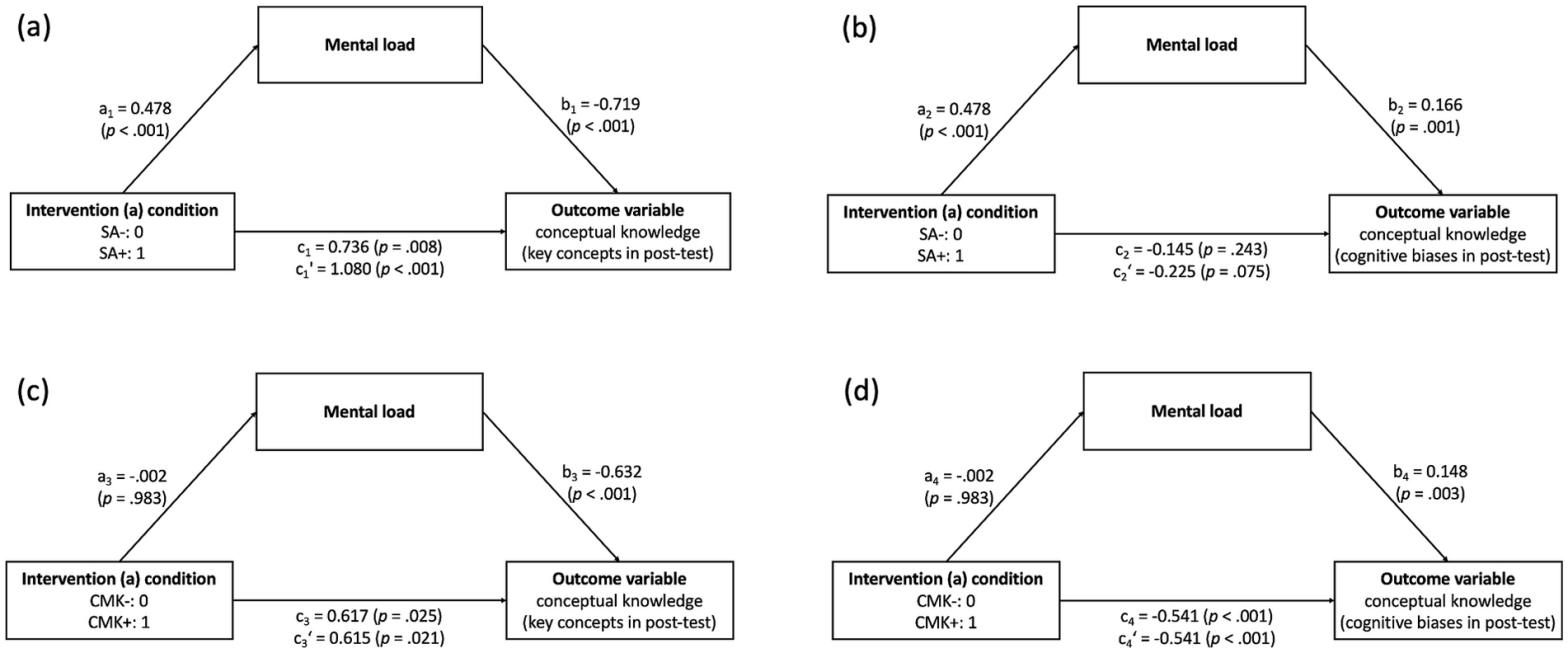
Models of intervention conditions as predictors of conceptual knowledge, mediated by mental load. **(a)** Mediation analysis with the intervention condition (with/without SA) as the predictor and use of key concepts in the post-test as the outcome variable. **(b)** Mediation analysis with the intervention condition (with/without SA) as the predictor and use of intuitive conceptions based on cognitive biases in the post-test as the outcome variable. **(c)** Mediation analysis with the intervention condition (with/without CMK) as the predictor and use of key concepts in the post-test as the outcome variable. **(d)** Mediation analysis with the intervention condition (with/without CMK) as the predictor and use of intuitive conceptions based on cognitive biases in the post-test as the outcome variable. a = effect of the predictor variable (i.e., the intervention condition) on the mediator (i.e., mental load); b = effect of the mediator on the outcome variable (i.e., conceptual knowledge); c’ = direct effect of the predictor variable on the outcome variable; c = total effect of the predictor variable on the outcome variable (a x b + c’); SA = intervention on self-assessment; CMK = instruction on conditional metaconceptual knowledge; plus sign (+) = the group received the respective intervention; minus sign (−) = the group did not receive the respective intervention.

We only found group differences in mental load but not in mental effort or self-efficacy (see 5.2.1 and 5.3.1); thus, mental effort and self-efficacy cannot mediate the influence of the interventions on conceptual knowledge but only mental load. Regarding intervention (a), we found a significant indirect effect of the self-assessment on students’ use of key concepts in the post-test through the mental load students experienced during the self-assessment, a_1_ x b_1_ = −0.344, 95%-CI [−0.527, −0.186] (see Figure 6a). Congruently, there was a significant indirect effect of the self-assessment on students’ use of intuitive conceptions based on cognitive biases in the post-test through the mental load students experienced during the self-assessment, a_2_ x b_2_ = 0.079, 95%-CI [0.028, 0.145] (see Figure 6b). In both models, the self-assessment increased students’ mental load, which then, in turn, decreased the number of key concepts, respectively, increased the number of intuitive conceptions based on cognitive biases students used in the post-test. Thus, the results indicate that the beneficial effects of the self-assessment (see Figure 6a for positive, direct effects on students’ use of key concepts in the post-test) were partially suppressed by the increased mental load.

In contrast, the mental load students experienced during intervention (b) – i.e., the instruction on conditional metaconceptual knowledge – did not mediate the influence of the respective intervention on students’ use of key concepts (indirect effect a_3_ x b_3_ = 0.001, 95%-CI [−0.127, 0.132]; see Figure 6c) or intuitive conceptions based on cognitive biases (indirect effect a_4_ x b_4_ = 0.000, 95%-CI [−0.034, 0.031]; see Figure 6d).

#### 5.3.3 RQ5: effects of students’ preconditions on cognitive load during the interventions

Students’ prior conceptual knowledge and general metaconceptual awareness and regulation (as reported in the pre-test) were partly negatively correlated with their mental load and positively with their mental effort during both interventions (for details, see Table 2).

**Table 2:** Correlational analyses between prior conceptual knowledge, metaconceptual awareness and regulation, and cognitive load during the interventions.

Variable	Prior conceptual knowledge (key concepts)	Prior conceptual knowledge (cognitive biases)	Metaconceptual awareness	Metaconceptual regulation
Mental load				
Intervention (a): SA+	−0.253*	0.091	−0.262*	−0.154*
Intervention (b): CMK+	−0.236*	0.069	−0.235*	−0.114
Mental effort				
Intervention (a): SA+	0.052	−0.003	0.069	0.165*
Intervention (b): CMK+	−0.017	−0.015	0.070	0.142*

Correlation coefficients based on two-tailed Spearman correlations; * = *p* < 0.05. Pairwise deletion. Only participants who received the respective interventions were included in the individual analyses. For the exact *p*-values and confidence intervals, see Supplementary Table S6, 7. SA+ = intervention on self-assessment; CMK+ = instruction on conditional metaconceptual knowledge.

## 6 Discussion

### 6.1 RQs concerning self-efficacy

Self-efficacy was positively influenced throughout the intervention phase, independent of whether students were in the intervention or control groups. This indicates that self-regulatory and metacognitive instruction focusing on students’ (intuitive) conceptions does not influence students’ self-efficacy differently compared to instruction only focused on subject-specific knowledge. Thus, when implementing self-regulatory and metacognitive instruction regarding student conceptions, instructors do not need to fear that confronting students with their intuitive conceptions will reduce their self-efficacy. Kirbulut and Uzuntiryaki-Kondakci (2019) suggested that metacognitive instruction regarding student conceptions could even be superior to traditional instruction in developing students’ self-efficacy. Our data could not support this hypothesis. However, our intervention phase was rather short, and students may need to engage more frequently in activities focusing on their conceptions on a metacognitive level to see progress in their conceptual knowledge. This may also be the reason for the decrease in self-efficacy from post-to follow-up test across all groups, as students most probably did not engage in activities promoting metaconceptual thinking and often not even in activities regarding the topic of evolution in school between both measuring points, leaving no chance for gaining a sense of mastery experience that could have promoted self-efficacy.

Regarding the self-assessment, there was no difference in self-efficacy between the groups. While according to a meta-analysis, self-assessments have an average positive effect on self-efficacy (Panadero et al., 2017), there are large differences in how self-assessments are operationalized (Andrade, 2019; Brown and Harris, 2013), and there are also studies reporting no effects (for a review, see also Panadero and Jonsson, 2013). Possible explanations for these divergent results include differences in the study design (e.g., the inclusion or non-inclusion of feedback), the duration of the self-assessment (e.g., recurring or single self-assessments), and the content of the self-assessment (e.g., writing skills versus one’s conceptions). Panadero et al. (2017) suggested that self-efficacy can be increased through criteria-referenced self-assessments because, firstly, providing students with information about the learning goals can make students feel more confident in being able to reach these goals. Secondly, students can see through repeated self-assessments that they become more competent over time and, thus, gain a sense of mastery experience. Thirdly, performance likely improves through self-assessments, which in turn increases students’ self-efficacy. While the latter may be applied to our study (see positive, direct effects of the self-assessment on students’ use of key concepts in the post-test in the mediation analyses), the other factors only apply to a limited extent to our study since the self-assessment was only done once and the control groups also received parts of the criteria (i.e., the list of key concepts) and thus information about the learning goals, which may have increased the control groups’ self-efficacy, too.

Regarding the instruction on conditional metaconceptual knowledge, there was no difference in self-efficacy between students in the intervention and control groups. Self-efficacy increased equivalently in all groups. In the control groups, this may be because of the acquired additional subject-specific knowledge. In the intervention groups, instruction on conditional metaconceptual knowledge seems to have been perceived as helpful in self-regulating intuitive conceptions based on cognitive biases in the context of evolution and consequently resulted in a positive self-efficacy development regarding explaining evolutionary changes scientifically appropriate. Theoretically, explicitly addressing students’ intuitive conceptions could have also negatively affected self-efficacy. However, valuing students’ intuitive conceptions in various contexts (e.g., in everyday life) during the instruction on conditional metaconceptual knowledge may have prevented detrimental effects. Thus, the approach of instruction on conditional metaconceptual knowledge may differ considerably from other approaches that deal with students’ intuitive conceptions, such as conceptual change approaches intending to provoke cognitive conflict and possibly negatively affect self-efficacy due to the dissatisfaction they aim to create (Pacaci et al., 2023).

Although the groups did not differ in their self-efficacy, they did differ in their self-efficacy bias. The analyses indicate that the groups without instruction on conditional metaconceptual knowledge strongly overestimated their capabilities after the intervention phase. In contrast, the groups with instruction on conditional metaconceptual knowledge developed more realistic calibrated self-efficacy beliefs. Even though we used a social reference (i.e., the average pre-test student) and not an external reference to determine under-and over-efficaciousness, we argue that the determined over-efficaciousness actually reflects over-efficaciousness because the average pre-test student most probably is already over-efficacious (Chen and Zimmerman, 2007; Klassen, 2002; Talsma et al., 2019; Zimmerman et al., 2011). While all groups developed similar self-efficacy levels through the intervention phase, they demonstrated different levels of conceptual knowledge after the intervention phase. For the groups with instruction on conditional metaconceptual knowledge, the gap between self-efficacy beliefs and actual knowledge was smaller. Our findings and interpretations are consistent with a study by Zimmerman et al. (2011), where self-regulatory training also did not increase students’ self-efficacy but their self-efficacy calibration. We argue that the instruction on conditional metaconceptual knowledge may have reduced students’ self-efficacy bias because students became metacognitively aware of the complex requirements of the task of explaining evolutionary changes, especially to avoid inappropriate intuitive conceptions. In the intervention, students learned on a metacognitive level about intuitive ways of thinking, to what extent the appropriateness of intuitive thinking is dependent on the context, and how intuitive thinking can result in inappropriate conceptions in the context of evolution. This may have allowed for a more accurately calibrated self-efficacy compared to the control groups that were not made metacognitively aware of intuitive ways of thinking and, consequently, may have developed biased self-efficacy beliefs because they continued to be unaware of flaws in their thinking within the context of evolution. Previous studies indicate that without self-regulatory and metacognitive instruction regarding student conceptions, students’ beliefs of their evolutionary understanding and their actual evolutionary understanding are highly disconnected (Schauer et al., 2014; Yates and Marek, 2014). For example, Yates and Marek (2014) reported that after instruction, students had even more inappropriate conceptions of evolution than prior to instruction but reported a higher confidence in their evolutionary understanding. It is crucial to reduce this over-efficaciousness – e.g., through instruction on conditional metaconceptual knowledge – since it can negatively impact self-regulation and performance (Talsma et al., 2019). Realistic self-efficacy, in contrast, may positively influence self-regulation and performance because realistic self-efficacy reflects accurate self-monitoring, which enables students to exert metacognitive control to regulate their strategies and knowledge, and within the specific context, their conceptions (Bol and Hacker, 2012; Chen, 2003; Stone, 2000; Talsma et al., 2019). Thus, even though the interventions did not increase students’ self-efficacy, at least the instruction on conditional metaconceptual knowledge led to a better fit between self-efficacy beliefs and capabilities by reducing students’ over-efficaciousness, with potentially positive effects on future learning and performance (see also Dunlosky and Rawson, 2012).

### 6.2 RQs concerning cognitive load

Regarding the effects of the interventions on cognitive load, we found no individual effect of the instruction on conditional metaconceptual knowledge but an individual effect of the self-assessment. The self-assessment required more cognitive capacity (i.e., mental load) than the control learning materials. Prior studies have reported inconclusive effects of self-assessments on cognitive load (e.g., Baars et al., 2014; Fastré et al., 2012; Krebs et al., 2022; Panadero and Romero, 2014), but the comparability is limited because of different designs of intervention and control groups and different content that is self-assessed. For example, Krebs et al. (2022) compared self-assessments with and without a rubric (and found a lower perceived difficulty reported by students using the rubric), while we compared a criteria-referenced self-assessment with a criteria-list without self-assessment. Thus, the findings are not directly comparable. Reasons for the effect on students’ mental load found in our study could be (1) that the process of the self-assessment itself is cognitively demanding, especially when students do not engage in self-assessments regularly, (2) that one’s conceptions as the self-assessed content are particularly cognitively demanding, and (3) that a considerable amount of the students did not have instruction on evolution before and thus low prior knowledge, and we found a negative correlation between prior conceptual knowledge and mental load (see also Dong et al., 2020; Kastaun et al., 2021; Seufert, 2018; Sweller et al., 1998). Consequently, the processing of the criteria of the self-assessment (i.e., the intuitive and scientific conceptions) and the task of performing a self-assessment itself may have required a high amount of mental load, leaving less cognitive resources for metacognitively engaging with one’s conceptions. This conclusion is supported by the results of the mediation analyses, demonstrating a negative effect of the mental load during the self-assessment activity on conceptual learning. However, students’ conceptual knowledge still improved through the self-assessment despite the higher mental load. However, if mental load could be reduced, gains in conceptual knowledge might be even higher.

Contrary to the self-assessment, the instruction on conditional metaconceptual knowledge did not influence students’ mental load individually, and mental load did not mediate the influence of the intervention on students’ conceptual knowledge. Hypothetically, it could also have been plausible that the instruction on conditional metaconceptual knowledge increased mental load because students were confronted with intuitive ways of thinking that are traditionally not part of science instruction and thus, led to the processing of more information compared to traditional instruction focusing on scientific conceptions only. However, the design of the instructional materials (i.e., a mix of informational texts and tasks) was similar to the design of the materials for the control groups and seemed to have prevented too high mental load. Interestingly, the group that did the self-assessment before the instruction on conditional metaconceptual knowledge (group SA + CMK+) reported significantly lower mental load during the second intervention (b) than the control group (SA-CMK-). An explanation could be that the acquired metaconceptual knowledge during the self-assessment reduced mental load during the instruction on conditional metaconceptual knowledge as students already engaged with their conceptions in the prior activity. This hypothesis is supported by the finding that self-reported metaconceptual awareness and regulation and mental load were negatively correlated, suggesting that students who frequently think about and regulate their conceptions perceive a lower mental load when working on activities on a metaconceptual level.

Contrary to mental load, both interventions did not affect mental effort. Since mental effort does not directly relate to the difficulty of a task (unlike mental load) but to the cognitive capacity a learner invests in performing a task (Krell, 2017; Paas and Van Merriënboer, 1994; Sweller et al., 2011), it is at least partly dependent on students’ motivation to invest effort in a task (Feldon et al., 2019; Krebs et al., 2022; Moreno, 2010; Schnotz et al., 2009). Thus, our results (i.e., a lack of differences between the groups) suggest (1) that even the mental load-increasing self-assessment did not reach such a high level of difficulty that students were prevented from investing effort in this task because they perceived it as a waste of energy and (2) that the mental load-decreasing activities did not reach such a low difficulty level that students perceived it unnecessary to invest effort for completing the activities (see also Feldon et al., 2019; Yeo and Neal, 2008). Similarly, there was no correlation between students’ prior conceptual knowledge and their mental effort during the self-regulatory and metacognitive instruction. Thus, the activities’ difficulty level also does not seem to have undermined the motivation to invest effort of students with differing prior knowledge.

Further, we found that self-reported general metaconceptual regulation (but not metaconceptual awareness) was positively related to the mental effort students invested in the self-regulatory and metacognitive instruction regarding student conceptions. This correlation may exist because students who habitually regulate their conceptions know from experience how much effort is needed for activities on a metaconceptual level and consequently self-regulate their effort investment, whereas merely being aware of one’s conceptions without performing actions related to one’s conceptions may not be enough to inform students about necessary effort investment. Generally, regulation requires effort investment, whereas awareness is not dependent on the willingness to invest cognitive capacities.

### 6.3 Limitations

This study has several strengths, such as the randomized experimental design, ecological validity through administration in the classroom, and large sample size. However, some limitations should also be considered. One of the limitations relates to the generalizability of the findings. While we included students from different school types and different regions (e.g., rural and urban areas) to get a heterogenous sample as representative as possible of German secondary education school students, we used convenience sampling. That means that the study conduction was dependent on teachers’ willingness to participate with their courses. Thus, we cannot generalize our findings to other educational levels, cultures, or topics other than evolution. However, we suggest that our findings and the investigated self-regulatory and metacognitive approaches regarding student conceptions are relevant beyond the specific context of this study and can be transferred to other target groups and scientific topics.

We acknowledge that another limitation of the presented study is that we cannot attribute the development in self-efficacy during the intervention phase to specific factors. For example, between measurement times one and two, students worked on both an interactive simulation and the first intervention materials (b: with/without self-assessment), and both or only one of the activities could have increased self-efficacy. However, due to the experimental design of our study, we could compare the groups that did or did not self-assess their conceptions (and the groups that did or did not receive instruction on conditional metaconceptual knowledge) and found no effect of both interventions on students’ self-efficacy. It should be noted that the aim of the instructional approaches was not to enhance self-efficacy. Instead, we investigated the effects of the instructional approaches on students’ self-efficacy as an exploratory question. Thus, the lack of significant results regarding self-efficacy is not a limitation of our study. Rather, this is an important finding concerning the effects of self-regulatory and metacognitive approaches regarding student conceptions on students’ self-efficacy as an important affective factor within (conceptual) learning. If researchers and instructors aim to find ways to promote self-efficacy (e.g., of under-efficacious students), it is suggested to base interventions on social cognitive theory and investigate single instructional factors derived from the main sources of self-efficacy in experimental intervention studies (van Dinther et al., 2011). This could be, for example, various forms of feedback, which is part of verbal persuasion as one of the main sources of self-efficacy.

Other limitations are related to the measurement of cognitive load. For example, we did not ask the participants about their cognitive load when applying their knowledge in the post-test. As a follow-up question, it would be worth investigating whether self-regulating one’s intuitive thinking (e.g., in the post-test) influences cognitive load. Additionally, in our study, we measured students’ cognitive load only after the complete interventions, i.e., after the self-assessment and after the instruction on conditional metaconceptual knowledge. This was done to avoid fatigue occurrence and reduce the time for completing the questionnaires. In future studies, cognitive load could be measured in a more fine-grained way. For example, each scientific and intuitive conception could be self-assessed individually to determine whether self-assessing certain types of conceptions, such as intuitive conception, may be cognitively more demanding than others. Further, measuring cognitive load in our study with the distinction between mental effort and mental load does not allow for making statements about whether the cognitive load induced by the learning materials was positive or negative to learning. However, measures that differentiate between different types of load, such as intrinsic, extraneous, and germane load, have been found to produce inconsistent results because learners often cannot accurately attribute their perceived difficulty of a task to a specific source (Schnotz and Kürschner, 2007; Sweller et al., 2011). For example, they struggle with the correct attribution to the intrinsic nature of the learning material (i.e., intrinsic load) or the way the learning material is presented (i.e., extraneous load). However, by investigating the mediating effect of mental load on the influence of the interventions on conceptual knowledge in the post-test, we were able to find in which direction mental load influenced learning.

### 6.4 Implications

The findings presented here have important implications for educational practice and research. While the findings are generally encouraging for implementing self-regulatory and metacognitive instruction regarding student conceptions in teaching, further research should investigate how students’ cognitive load during a self-assessment of one’s conceptions can be adjusted to make this instructional approach even more effective. For example, as the correlational analyses suggest, more prior (meta-)conceptual knowledge may reduce cognitive load, and students could learn more about the topic and intuitive conceptions on a metaconceptual level before being asked to self-assess their conceptions. Further, repeated self-assessments of one’s conceptions may reduce cognitive load because students internalize the criteria and proceduralize their skills and may, as a side-effect, also increase accurately calibrated self-efficacy because students observe the changes in their conceptions in the respective context (see also Panadero et al., 2017; Wirth et al., 2020). With this, of course, the purpose of the self-assessment would change from becoming metaconceptually aware of one’s conceptions to metaconceptually monitoring the regulation of one’s conceptions continuously, which could also positively influence students’ self-regulatory skills and conceptual knowledge acquisition. Regardless of the effect of the self-assessment activity on mental load, both the self-assessment of one’s conceptions and the instruction on conditional metaconceptual knowledge should be implemented in educational contexts because both approaches have been found to increase conceptual knowledge (see also Hartelt and Martens, 2024a) and more accurately calibrated self-efficacy. We suggest that the instructional approaches examined in this study can be transferred to other contexts. The instructional approaches may be especially relevant to other scientific topics where intuitive conceptions can also be obstacles to conceptual knowledge acquisition (Coley et al., 2017; Coley and Tanner, 2012). However, future research is needed. Besides the specific self-regulatory and metacognitive approaches investigated in this paper, fellow researchers may want to investigate the relationship between other components and phases of self-regulated learning on the one side and self-efficacy and cognitive load on the other side. For example, while we have investigated the relationship between self-assessments (as part of the self-reflection phase of self-regulated learning) and cognitive load, future research may investigate the relationship between the use of self-control and self-observation strategies in relation to one’s conceptions during the performance phase and students’ cognitive load.

## 7 Conclusion

This study has contributed to understanding how instructional approaches based on self-regulated learning and metacognition, intended to make students metacognitively aware of their conceptions and support them to self-regulate their conceptions context-dependently, influence affective and cognitive factors (i.e., self-efficacy and cognitive load). The findings suggest that becoming metacognitively aware of one’s (intuitive) conceptions does not affect one’s self-efficacy negatively. Instead, acquiring metacognitive/metaconceptual knowledge is helpful for accurate self-efficacy calibration, underlining the importance of metacognition for learning as accurate beliefs about one’s abilities are essential for self-regulated learning. Cognitive load was affected differently by the investigated self-regulatory and metacognitive approaches, demonstrating the need for research to consider cognitive load individually for different instructional approaches, even when they share essential common features (e.g., addressing intuitive thinking). In this study, a self-assessment of one’s conceptions increased mental load (in contrast to instruction on conditional metaconceptual knowledge), resulting in suppressing effects on the acquisition of conceptual knowledge. Students’ general metaconceptual awareness and regulation were negatively correlated with mental load, thus implying that continuously promoting students’ metaconceptual thinking may reduce cognitive load during student conception-focused self-regulatory and metacognitive instruction (such as a self-assessment of one’s conceptions). Thus, it is suggested to regularly implement self-regulatory and metacognitive instruction focusing on students’ conceptions in teaching.

## Data Availability

The raw data supporting the conclusions of this article will be made available by the authors, without undue reservation.

## Footnotes

^1^ https://www.uni-kassel.de/go/simulationevolution
