# Supplementary material for: Self-regulatory and metacognitive instruction regarding student conceptions: influence on students’ self-efficacy and cognitive load
Source: Front Psychol. 2024 Oct 22;15:1450947. doi: 10.3389/fpsyg.2024.1450947 (PMC11534677; doi:10.3389/fpsyg.2024.1450947)
Supplement: Supplementary file 1 [file Table_1.docx]

Supplementary Material

# Supplementary Table 1

Descriptive statistics of the intervention groups and total sample: Pre-test performance scores and control variables

| Variable | Group: SA+CMK+ | | | Group: SA+CMK- | | | Group: SA-CMK+ | | | Group: SA-CMK- | | | Total sample | | |
| --- | --- | --- | --- | --- | --- | --- | --- | --- | --- | --- | --- | --- | --- | --- | --- |
|  | *n* | *M* | *SD* | *n* | *M* | *SD* | *n* | *M* | *SD* | *n* | *M* | *SD* | *n* | *M* | *SD* |
| Self-efficacy ^a^ | 152 | 3.00 | 1.02 | 140 | 3.17 | 1.04 | 152 | 3.09 | 1.03 | 143 | 3.19 | 0.99 | 587 | 3.11 | 1.02 |
| Conceptual knowledge ^b^  Key concepts  Cognitive biases | 153  153 | 2.95  2.10 | 3.04  1.66 | 144  144 | 3.29  2.49 | 2.84  1.62 | 158  158 | 3.20  2.07 | 3.03  1.47 | 146  146 | 3.21  2.19 | 3.13  1.63 | 601  601 | 3.16  2.21 | 3.01  1.60 |
| NSPQ | 153 | 0.53 | 0.31 | 144 | 0.57 | 0.28 | 158 | 0.55 | 0.29 | 146 | 0.55 | 0.30 | 601 | 0.55 | 0.29 |
| Metaconceptual awareness ^a^ | 150 | 4.52 | 0.79 | 141 | 4.48 | 0.84 | 154 | 4.39 | 0.82 | 145 | 4.49 | 0.80 | 590 | 4.47 | 0.81 |
| Metaconceptual regulation ^a^ | 150 | 3.72 | 0.85 | 140 | 3.69 | 0.92 | 154 | 3.72 | 0.84 | 146 | 3.76 | 0.94 | 590 | 3.72 | 0.88 |
| Biology grade ^c^ | 147 | 10.01 | 2.76 | 136 | 10.10 | 2.66 | 151 | 9.63 | 2.80 | 144 | 10.06 | 2.85 | 578 | 9.94 | 2.77 |
| Age | 149 | 17.22 | 1.08 | 136 | 17.29 | 1.12 | 151 | 17.38 | 1.05 | 142 | 17.26 | 1.15 | 578 | 17.29 | 1.09 |
| Class level  10  11  12  13 | 17  30  55  48 |  |  | 11  28  52  46 |  |  | 11  33  60  48 |  |  | 16  34  56  38 |  |  | 55  125  223  180 |  |  |
| Gender  Male  Female  Diverse | 52  96  1 |  |  | 43  91  3 |  |  | 49  101  1 |  |  | 52  88  4 |  |  | 196  376  9 |  |  |
| Upper secondary school  Gymnasium  Comprehensive school | 120  30 |  |  | 113  24 |  |  | 120  32 |  |  | 116  28 |  |  | 469  114 |  |  |
| Course type  Basic  Advanced | 77  72 |  |  | 68  69 |  |  | 75  77 |  |  | 76  68 |  |  | 296  286 |  |  |
| First language  German  Other | 114  29 |  |  | 115  21 |  |  | 123  21 |  |  | 105  28 |  |  | 457  99 |  |  |
| Previous evolution instruction before upper secondary level  Yes  No | 82  68 |  |  | 82  52 |  |  | 91  60 |  |  | 76  66 |  |  | 331  246 |  |  |
| Number of evolution lessons in upper secondary level ^d^ | 140 | 9.70 | 13.12 | 125 | 9.06 | 11.77 | 143 | 9.92 | 12.97 | 136 | 10.95 | 16.38 | 544 | 9.92 | 13.67 |

*Note.* The table is based on the sample of *N* = 602 students who worked at least on the pre-test, intervention materials, the tests after the interventions, and the post-test. Due to missing data, numbers may not add up to 602 for individual variables. SA = intervention on self-assessment; CMK = instruction on conditional metaconceptual knowledge; plus sign (+) = the group received the respective intervention; minus sign (-) = the group did not receive the respective intervention.

^a^ Self-efficacy, metaconceptual awareness, and metaconceptual regulation were measured on six-point Likert scales ranging from 1 = low to 6 = high. ^b^ Conceptual knowledge was measured by adding the number of key concepts (possible range: 0–14) and cognitive biases (possible range: 0–6) used in both ACORNS items. ^c^ Biology grade was measured within the possible range of 0 = insufficient performance and 15 = very good performance. ^d^ 98% Winsorization.
