# Supplementary material for: Self-regulatory and metacognitive instruction regarding student conceptions: influence on students’ self-efficacy and cognitive load
Source: Front Psychol. 2024 Oct 22;15:1450947. doi: 10.3389/fpsyg.2024.1450947 (PMC11534677; doi:10.3389/fpsyg.2024.1450947)
Supplement: Supplementary file 2 [file Table_2.docx]

Supplementary Material

# Supplementary Table 2

Natural selection performance quotient scores

| Scientific key concepts | Intuitive conceptions based on cognitive biases | NSPQ |
| --- | --- | --- |
| 0 | 0 | 0.00 |
| 0 | 1 | 0.00 |
| 0 | 2 | 0.00 |
| 0 | 3 | 0.00 |
| 1 | 0 | 0.70 |
| 1 | 1 | 0.51 |
| 1 | 2 | 0.43 |
| 1 | 3 | 0.39 |
| 2 | 0 | 0.80 |
| 2 | 1 | 0.64 |
| 2 | 2 | 0.56 |
| 2 | 3 | 0.50 |
| 3 | 0 | 0.86 |
| 3 | 1 | 0.72 |
| 3 | 2 | 0.64 |
| 3 | 3 | 0.59 |
| 4 | 0 | 0.90 |
| 4 | 1 | 0.79 |
| 4 | 2 | 0.71 |
| 4 | 3 | 0.65 |
| 5 | 0 | 0.94 |
| 5 | 1 | 0.83 |
| 5 | 2 | 0.76 |
| 5 | 3 | 0.71 |
| 6 | 0 | 0.97 |
| 6 | 1 | 0.88 |
| 6 | 2 | 0.81 |
| 6 | 3 | 0.75 |
| 7 | 0 | 1.00 |
| 7 | 1 | 0.91 |
| 7 | 2 | 0.84 |
| 7 | 3 | 0.79 |

*Note.* The NSPQ (natural selection performance quotient) was calculated based on the suggestions by Nehm & Reilly (2007). According to Nehm & Reilly (2007), “the NSPQ takes a ratio of key-concept diversity to the sum of key-concept diversity and misconception diversity, multiplies it by the ratio of key-concept diversity to total possible key concepts, and produces a single performance score on a 0 to 100, gradelike scale. The first term expresses the proportion of the students’ answers that were correct, and the second expresses how the correct proportion compared to the most complete possible answer. Exponents were chosen to calibrate the NSPQ scale so that it conformed to our assessment that four key concepts would result in a score greater than 65. In addition to permitting the visualization of student knowledge on a single scale, the NSPQ also distinguishes clearly between students who have problems with their understanding of natural selection, despite displaying significant knowledge, and those students with no misconceptions who displayed differing levels of knowledge” (p. 266). However, building on these authors’ work, we interpret one cognitive bias as two “misconceptions” to calculate the NSPQ, because cognitive biases are at a broader level than the specific “misconceptions” originally used to calculate the NSPQ.

Nehm, R. H., & Reilly, L. (2007). Biology majors’ knowledge and misconceptions of natural selection. *BioScience*, *57*(3), 263–272.
