# Supplementary material for: Self-regulatory and metacognitive instruction regarding student conceptions: influence on students’ self-efficacy and cognitive load
Source: Front Psychol. 2024 Oct 22;15:1450947. doi: 10.3389/fpsyg.2024.1450947 (PMC11534677; doi:10.3389/fpsyg.2024.1450947)
Supplement: Supplementary file 3 [file Table_3.docx]

# *Supplementary Material*

# Supplementary Table 3

Development of students’ self-efficacy (mean scores ± standard deviations) over the different measuring points as a function of group allocation

| Self-efficacy | Group | | | |
| --- | --- | --- | --- | --- |
|  | SA+CMK+ | SA+CMK- | SA-CMK+ | SA-CMK- |
| Pre-test | 3.01 ± 1.03 | 3.28 ± 1.03 | 2.91 ± 1.05 | 3.30 ± 1.01 |
| After intervention (a) with/without self-assessment | 3.84 ± 0.89 | 3.93 ± 0.87 | 3.82 ± 1.02 | 4.10 ± 0.95 |
| After intervention (b) with/without instruction on conditional metaconceptual knowledge | 4.06 ± 0.86 | 4.17 ± 0.93 | 4.00 ± 1.04 | 4.24 ± 0.86 |
| Follow-up test | 3.67 ± 0.98 | 3.84 ± 0.93 | 3.52 ± 1.08 | 3.81 ± 0.92 |

# *Note.* SA = intervention on self-assessment; CMK = instruction on conditional metaconceptual knowledge; plus sign (+) = the group received the respective intervention; minus sign (-) = the group did not receive the respective intervention.
