# Supplementary material for: Self-regulatory and metacognitive instruction regarding student conceptions: influence on students’ self-efficacy and cognitive load
Source: Front Psychol. 2024 Oct 22;15:1450947. doi: 10.3389/fpsyg.2024.1450947 (PMC11534677; doi:10.3389/fpsyg.2024.1450947)
Supplement: Supplementary file 4 [file Table_4.docx]

# *Supplementary Material*

# Supplementary Table 4

Development of students’ self-efficacy bias (mean scores ± standard deviations) over the different measuring points as a function of group allocation

| Self-efficacy bias | Group | | | |
| --- | --- | --- | --- | --- |
|  | SA+CMK+ | SA+CMK- | SA-CMK+ | SA-CMK- |
| Pre-test | -0.04 ± 1.13 | 0.26 ± 1.05 | -0.09 ± 1.21 | 0.16 ± 1.09 |
| After the intervention phase (a and b) | 0.56 ± 1.12 | 0.89 ± 1.20 | 0.67 ± 1.05 | 1.08 ± 0.94 |
| Follow-up test | -0.01 ± 1.04 | 0.41 ± 1.14 | 0.14 ± 0.93 | 0.43 ± 1.02 |

# *Note.* Values above zero reflect over-efficaciousness and values below zero reflect under-efficaciousness (in comparison to average pre-test efficaciousness). Self-efficacy bias was only determined for the three measurement points depicted above but not after intervention (a) because after intervention (a), only self-efficacy was measured but not conceptual knowledge. SA = intervention on self-assessment; CMK = instruction on conditional metaconceptual knowledge; plus sign (+) = the group received the respective intervention; minus sign (-) = the group did not receive the respective intervention.
