# Supplementary material for: Self-regulatory and metacognitive instruction regarding student conceptions: influence on students’ self-efficacy and cognitive load
Source: Front Psychol. 2024 Oct 22;15:1450947. doi: 10.3389/fpsyg.2024.1450947 (PMC11534677; doi:10.3389/fpsyg.2024.1450947)
Supplement: Supplementary file 5 [file Table_5.docx]

***Supplementary Material***

**Supplementary Table 5**

Students’ cognitive load related to the interventions (mean scores ± standard deviations)

| Variable | Group | | | |
| --- | --- | --- | --- | --- |
|  | SA+CMK+ | SA+CMK- | SA-CMK+ | SA-CMK- |
| Mental load |  |  |  |  |
| Related to the intervention (a) with/without self-assessment | 3.24 ± 1.22 | 3.07 ± 1.20 | 2.60 ± 1.08 | 2.79 ± 1.27 |
| Related to the intervention (b) with/without instruction on conditional metaconceptual knowledge | 3.12 ± 1.21 | 3.26 ± 1.16 | 3.37 ± 1.29 | 3.53 ± 1.26 |
| Mental effort |  |  |  |  |
| Related to the intervention (a) with/without self-assessment | 4.59 ± 1.14 | 4.59 ± 1.22 | 4.74 ± 1.30 | 4.80 ± 1.30 |
| Related to the intervention (b) with/without instruction on conditional metaconceptual knowledge | 4.71 ± 1.27 | 4.68 ± 1.27 | 4.78 ± 1.27 | 4.99 ± 1.26 |

# *Note.* SA = intervention on self-assessment; CMK = instruction on conditional metaconceptual knowledge; plus sign (+) = the group received the respective intervention; minus sign (-) = the group did not receive the respective intervention.
