# Supplementary material for: Self-regulatory and metacognitive instruction regarding student conceptions: influence on students’ self-efficacy and cognitive load
Source: Front Psychol. 2024 Oct 22;15:1450947. doi: 10.3389/fpsyg.2024.1450947 (PMC11534677; doi:10.3389/fpsyg.2024.1450947)
Supplement: Supplementary file 6 [file Table_6.docx]

# *Supplementary Material*

# Supplementary Table 6

Correlational analyses between prior conceptual knowledge and cognitive load during the interventions

| Variable | Prior conceptual knowledge  (key concepts) | | | | | Prior conceptual knowledge  (cognitive biases) | | | | |
| --- | --- | --- | --- | --- | --- | --- | --- | --- | --- | --- |
|  | *r_s_* | 95% CI | | *p* | *n* | *r_s_* | 95% CI | | *p* | *n* |
|  |  | *LL* | *UL* |  |  |  | *LL* | *UL* |  |  |
| Mental load |  |  |  |  |  |  |  |  |  |  |
| Intervention (a): SA+ | -.253 | -.365 | -.140 | < .001 | 280 | .091 | -.026 | .204 | .128 | 280 |
| Intervention (b): CMK+ | -.236 | -.339 | -.130 | < .001 | 281 | .069 | -.048 | .187 | .232 | 281 |
| Mental effort |  |  |  |  |  |  |  |  |  |  |
| Intervention (a): SA+ | .052 | -.068 | .170 | .381 | 282 | -.003 | -.122 | .128 | .963 | 282 |
| Intervention (b): CMK+ | -.017 | -.140 | .109 | .777 | 281 | -.015 | -.138 | .105 | .807 | 281 |

*Note.* Two-tailed Spearman correlations. Pairwise deletion. Only participants who received the respective interventions were included in the individual analyses. CI = confidence interval; LL = lower limit; UL = upper limit; SA+ = intervention on self-assessment; CMK+ = instruction on conditional metaconceptual knowledge.
