# Supplementary material for: Self-regulatory and metacognitive instruction regarding student conceptions: influence on students’ self-efficacy and cognitive load
Source: Front Psychol. 2024 Oct 22;15:1450947. doi: 10.3389/fpsyg.2024.1450947 (PMC11534677; doi:10.3389/fpsyg.2024.1450947)
Supplement: Supplementary file 7 [file Table_7.docx]

Supplementary Material

# Supplementary Table 7

Correlational analyses between metaconceptual awareness and regulation and cognitive load during the interventions

| Variable | Metaconceptual awareness | | | | | Metaconceptual regulation | | | | |
| --- | --- | --- | --- | --- | --- | --- | --- | --- | --- | --- |
|  | *r_s_* | 95% CI | | *p* | *n* | *r_s_* | 95% CI | | *p* | *n* |
|  |  | *LL* | *UL* |  |  |  | *LL* | *UL* |  |  |
| Mental load |  |  |  |  |  |  |  |  |  |  |
| Intervention (a): SA+ | -.262 | -.380 | -.141 | < .001 | 274 | -.154 | -.261 | -.032 | .011 | 273 |
| Intervention (b): CMK+ | -.235 | -.347 | -.119 | < .001 | 292 | -.114 | -.229 | .004 | .052 | 291 |
| Mental effort |  |  |  |  |  |  |  |  |  |  |
| Intervention (a): SA+ | .069 | -.058 | .199 | .254 | 276 | .165 | .045 | .290 | .006 | 275 |
| Intervention (b): CMK+ | .070 | -.051 | .186 | .236 | 292 | .142 | .017 | .261 | .015 | 292 |

*Note.* Two-tailed Spearman correlations. Pairwise deletion. Only participants who received the respective interventions were included in the individual analyses. CI = confidence interval; LL = lower limit; UL = upper limit; SA+ = intervention on self-assessment; CMK+ = instruction on conditional metaconceptual knowledge.
